# Correlation between symptom distress and quality of life in patients with Chronic Kidney Disease Stage 5 undergoing hemodialysis in China: a multi-center cross-sectional study

**DOI:** 10.3389/fpsyt.2026.1733502

**Published:** 2026-03-27

**Authors:** Xue Qiao, Yifei Wang, Zhaoyang Tian, Yongli Zhan, Tao Yang, Jie Luan, Erping Yan, Xiaojuan Li, Tang Tang, Qiang Ji, Tianxu Yang, Li Wang, Li Wan, Guangqin Mu, Yanhui Li, Shaohua Wang, Jin Qian, Lulu Liu, Pingping Sun, Rui Jing, Zhan Wang, Fangfang Kang, Yu Kang, Lu Xu, Jianying Hao, Yushan Yang, Yan Ren, Xingxing Pang, Shuo Bai, Yaxin Zhu, Yina He, Wen Zhang, Tianxu Zhang, Zhenze Han, Peng Liu, Moyan Qiu

**Affiliations:** 1 Wangjing Hospital, China Academy of Chinese Medical Sciences, Beijing, China; 2 Blood Purification Center, Wangjing Hospital, China Academy of Chinese Medical Sciences, Beijing, China; 3 Dialysis Unit, Fangshan Hospital, Beijing University of Chinese Medicine, Beijing, China; 4 Department of Nephrology, Guang’anmen Hospital, China Academy of Chinese Medical Sciences, Beijing, China; 5 Nephrology Rehabilitation Center, Beijing Zhongguancun Hospital, Beijing, China; 6 Department of Artificial Intelligence, Xiamen University, Xiamen, China; 7 School of Public Health, Sun Yat-sen University, Guangzhou, China; 8 Graduate School, Beijing University of Chinese Medicine, Beijing, China

**Keywords:** Chronic Kidney Disease, cross-sectional study, hemodialysis, quality of life, symptom distress

## Abstract

**Aim:**

To explore the symptom distress and quality of life status of patients with Chronic Kidney Disease (CKD) Stage 5 undergoing hemodialysis in China, and their correlation.

**Methods:**

The Dialysis Symptom Index (DSI) and the Kidney Disease Quality of Life Short Form (KDQOL-SF™ 1.3), two widely used assessment tools for hemodialysis patients, were used for evaluation.

**Results:**

The patients reported an average of 13 symptoms. The most common symptoms were worrying (99.7%), sexual dysfunction (>90%), and trouble falling asleep (90.8%). The KDQOL-SF™ 1.3 showed that patients had lower scores in dimensions such as “Burden of Kidney Disease” and “Work Status,” while they had higher scores in the “Dialysis Staff Encouragement” and “Role Limitations - Emotional” dimensions. Univariate analysis revealed that age, education level, employment status, dialysis frequency, dialysis modality, primary disease, multiple comorbidities, complications (renal anemia), and polypharmacy were significantly associated with the total DSI score. Spearman’s rank correlation analysis showed a negative correlation between the total DSI score and each dimension of KDQOL-SF™ 1.3, with the strongest correlation found with the “Symptom/Problem List” dimension. Multivariate linear regression analysis indicated that age, education level, dialysis duration, dialysis modality, and primary disease (diabetic nephropathy) were independent influencing factors for the total DSI score. After controlling for confounding factors, the total DSI score remained independently negatively correlated with multiple dimensions of KDQOL-SF™ 1.3.

**Conclusions:**

Symptom distress is negatively correlated with quality of life in Chinese CKD Stage 5 hemodialysis patients. Clinical attention should be given to symptom screening and management.

## 1 Introduction

Chronic Kidney Disease (CKD) has become an increasingly severe public health issue worldwide. According to the GBD 2021 data, the number of CKD patients worldwide in 2021 was approximately 673.7 million, accounting for about 8.54% of the global population (1). Compared to 1990, the absolute number of CKD cases has increased by nearly 92%. Although the age-standardized prevalence has slightly decreased, the overall disease burden has still risen significantly (1). According to a study by Deng et al. (2025), the number of deaths due to CKD in 2021 was approximately 1,527,639, with an age-standardized mortality rate (ASMR) of about 18.50 per 100,000 people (2). The disability-adjusted life years (DALYs) caused by CKD reached approximately 44,453,684 years in 2021, with an age-standardized DALYs rate of about 529.62 per 100,000 people (2). In CKD Stage 5 patients undergoing maintenance hemodialysis, these numbers suggest a significant symptom distress, with their quality of life severely impacted.

Hemodialysis is a long-term renal replacement therapy that begins with self-management and continues throughout the course of treatment. Its main goal is to remove toxins from the body and maintain water, electrolyte, and acid-base balance, thereby sustaining vital functions. At the same time, the effectiveness of dialysis and its long-term impacts are closely related to the patient’s quality of life, making it an important focus in both clinical practice and research (3). Long-term hemodialysis is a complex and ongoing treatment process that not only has a profound impact on the patient’s physical and psychological state but also significantly affects their family life and social functioning. Studies consistently show that the quality of life of hemodialysis patients is generally lower than that of the general population (4–6).

Research indicates that symptom distress in CKD Stage 5 hemodialysis patients is negatively correlated with their quality of life (7–11). Therefore, to improve the quality of life of hemodialysis patients, it is essential to accurately identify and assess their symptom distress, and to develop targeted interventions based on these assessments, along with continuous monitoring and follow-up evaluations.

Various assessment and measurement tools have been developed to evaluate symptom distress in CKD Stage 5 hemodialysis patients (12–16).

According to the World Health Organization (WHO), quality of life is defined as “an individual’s perception of their position in life within the context of the culture and value systems in which they live, and in relation to their goals, expectations, standards, and concerns” (17).

Assessing the relationship between symptom distress and quality of life in CKD Stage 5 hemodialysis patients has gradually become an important focus in clinical research and chronic disease management. Traditionally, the assessment of hemodialysis treatment efficacy has focused mainly on laboratory indicators, dialysis adequacy (such as Kt/V, URR), or survival outcomes (3). However, given that symptom distress is widespread and persistent in this population, incorporating patient-reported outcome measures (such as symptom distress and quality of life) is of significant importance for optimizing clinical management strategies and improving overall patient well-being (18).

This study uses the Dialysis Symptom Index (DSI) (12) to assess symptom distress in patients and the Kidney Disease Quality of Life Short Form (KDQOL-SF™ 1.3) (13, 19) to assess their quality of life.

Therefore, the aim of this study is to analyze the sociodemographic and disease-related characteristics of CKD Stage 5 hemodialysis patients admitted to blood purification centers in Chinese hospitals, and to explore the correlation between their symptom distress and quality of life.

## 2 Materials and methods

### 2.1 Sample and design

This study is a multi-center cross-sectional study. A consecutive sampling method was used to continuously recruit all patients who met the inclusion criteria at the blood purification centers of four hospitals: Wangjing Hospital, China Academy of Chinese Medical Sciences; Fangshan Hospital, Beijing University of Chinese Medicine; Guang’anmen Hospital, China Academy of Chinese Medical Sciences, and Beijing Zhongguancun Hospital, from March to September 2025. Researchers at each center conducted recruitment based on a unified investigator manual and standardized procedures.

Sample size calculation: According to the general principle of sample size estimation for multivariate analysis, the sample size should be at least 5 to 10 times the number of independent variables. This study plans to include a total of 29 independent variables after dummy variable transformation. Based on this, the estimated required sample size is 145 to 290 cases. Considering a 10% invalid questionnaire rate and sampling errors, the sample size should be increased to 160 to 319 cases. To ensure the robustness of the study and consider the actual capacity of each center, the final sample size was increased to 360 cases.

Inclusion criteria: ① Age ≥ 18 years; ② Meet the diagnostic criteria for CKD Stage 5; ③ Undergo regular hemodialysis for ≥ 3 months, with a dialysis frequency of 2 to 4 times per week; ④ Clear consciousness, with normal comprehension and expression abilities; ⑤ Voluntarily participate in the study, sign the informed consent form, and actively cooperate with the questionnaire survey.

Exclusion criteria: ① Comorbidity with severe cardiovascular and cerebrovascular diseases, acute infections, malignant tumors, severe gastrointestinal and liver diseases, fractures, or other conditions; ② Unable to cooperate with the survey or refuse to sign the informed consent form.

### 2.2 Ethical approval, informed consent, and data collection

The procedures of this study follow the relevant provisions of the Declaration of Helsinki (20) and the Oviedo Convention (21), and have been approved by the Medical Ethics Committee of Wangjing Hospital, China Academy of Chinese Medical Sciences (approval number: WJEC-KT-2024-071-P004).

The researchers provided all patients with a detailed explanation of the purpose of the study and obtained written informed consent from each participant. Data collection was conducted using the “CKD Stage 5 Data Platform” developed by Xiamen University (http://47.108.133.39:3000/login).

### 2.3 Survey tools

This study was conducted after patients received hemodialysis treatment at the blood purification center and their condition stabilized. All questionnaires were independently completed by the patients based on their subjective experiences.

Data collection process: Patients log into the “CKD Stage 5 Data Platform” using mobile devices for online self-assessment. For those who are unable to complete the questionnaire independently due to reasons such as arteriovenous fistula puncture treatment, severe vision impairment, or lack of proficiency with mobile devices, trained researchers read the questions and options aloud, and the patients respond orally. The researchers then record and mark the responses accurately. Family members, doctors, and nurses are not involved in evaluating or answering any items to ensure that all responses come directly from the patients themselves.

The survey questionnaire includes variables related to sociodemographic characteristics (gender, age, education level, marital status, occupation) and disease-related information (BMI, dialysis duration, dialysis frequency, Kt/V, URR, dialysis modality, primary disease, comorbidities, complications, and polypharmacy).

Measurement tools: This study used two measurement tools to assess patients. The first is the DSI, which aims to evaluate the presence and severity of common physical and emotional symptoms experienced by hemodialysis patients in the past week. The second is the Chinese version of the KDQOL-SF™ 1.3 scale, which is used to assess health-related quality of life in CKD patients.

1. DSI: This scale consists of 30 items, including physical symptoms (such as constipation, nausea, vomiting, diarrhea, itching, shortness of breath, muscle cramps) and emotional symptoms (such as feeling anxious, feeling irritable, difficulty concentrating). For each symptom, patients self-assess its presence and the level of distress it caused in the past week, using a 5-point Likert scale, ranging from 0 (not bothersome at all) to 4 (very bothersome).
2. KDQOL-SF™ 1.3: The scale consists of two parts: the SF-36 generic core dimensions and kidney disease-targeted dimensions, totaling 79 items, with standardized scores ranging from 0 to 100. The SF-36 generic core dimensions include 8 domains (such as physical functioning, emotional well-being, energy/fatigue, etc.), while the kidney disease-targeted dimensions include 11 dimensions: symptom/problem list, effects of kidney disease, burden of kidney disease, work status, cognitive function, quality of social interaction, sexual function, sleep, social support, dialysis staff encouragement, and patient satisfaction. This scale is widely used to assess quality of life in CKD patients, with higher scores indicating better quality of life (19).

### 2.4 Statistical analysis

Statistical analysis was performed using IBM SPSS Statistics version 31.0.

Categorical and qualitative variables were described using frequencies (n) and percentages (%), while continuous variables were described using the median, interquartile range, minimum, and maximum values.

The Wilcoxon rank-sum test was used to compare the differences in total DSI scores between two groups based on gender, marital status, employment status, dialysis frequency, dialysis modality, comorbidities, and complications. The Kruskal-Wallis H test was used to compare the differences in total DSI scores among multiple groups (education level, primary disease, and polypharmacy), with the Bonferroni method applied to correct the significance level for multiple comparisons. Spearman’s rank correlation analysis was used to assess the correlation between continuous variables such as age, BMI, dialysis duration, Kt/V, URR, and the total DSI score, and to analyze the association between the total DSI score and the scores of each dimension of KDQOL-SF™ 1.3.

The total DSI score was used as the dependent variable, and sociodemographic and disease-related variables were included in a multiple linear regression analysis to explore the independent factors affecting symptom distress. Additionally, the scores of each dimension of KDQOL-SF™ 1.3 were used as dependent variables, with the total DSI score as the core independent variable. The above-mentioned potential confounding factors were adjusted, and multiple linear regression models were constructed separately to assess the independent associations between symptom distress and the dimensions of quality of life.

In all comparisons, a p-value < 0.05 was considered statistically significant.

## 3 Results

### 3.1 Sociodemographic characteristics and disease-related data of the sample

During the 7-month study period, a total of 360 patients were screened. One patient was excluded during the review phase due to a dialysis duration of less than 3 months. Ultimately, 359 patients who met the inclusion criteria were included in the analysis. All included patients completed the study.

In the study sample, the proportion of males was higher than that of females (201 males, 56.0%; 158 females, 44.0%). The median age of the participants was approximately 60 years. Most patients (62.4%) had an education level of junior high school, high school, vocational school, or technical high school. The majority of patients were married (86.1%). Regarding employment status, non-employed individuals (55.7%) accounted for a higher proportion. The sociodemographic characteristics of the sample are detailed in **Table 1**.

**Table 1:** Sociodemographic characteristics of the sample (n = 359).

Variable		Sample
Gender, *n* (%)	Male	201 (56.0)
	Female	158 (44.0)
Age (years)	Median [Q1; Q3]	60 [51; 68]
	Minimum	25
	Maximum	89
Education, *n* (%)	Primary school and below	18 (5.0)
	Junior high school/High school/Vocational school/Technical high school	224 (62.4)
	Associate degree/Bachelor’s degree or above	117 (32.6)
Marital status, *n* (%)	Married	309 (86.1)
	Unmarried	50 (13.9)
Employment status, *n* (%)	Employed	159 (44.3)
	Not employed	200 (55.7)

Q1, First quartile; Q3, Third quartile.

The median BMI of the patients was 23.3 kg/m^2^, and the median dialysis duration was 58 months. The vast majority of patients (90.3%) received a regular dialysis regimen of 3 sessions per week. In terms of dialysis adequacy, the median Kt/V and urea reduction ratio (URR) were 1.3 and 67.2%, respectively. Conventional hemodialysis (54.0%) was the most common dialysis modality.

Chronic glomerulonephritis (29.5%), diabetic nephropathy (27.9%), and hypertensive kidney damage (23.4%) are the main primary diseases leading to CKD Stage 5. In terms of comorbidities, hypertension is the most common (50.7%).

The patients had a heavy burden of complications, with the highest prevalence of CKD-mineral and bone disorder (75.5%) and renal anemia (74.1%).

54.3% of patients were on polypharmacy, and 26.2% of patients had excessive polypharmacy. Disease-related information of the patients is detailed in **Table 2**.

**Table 2:** Disease-related information of the patients (n = 359).

Variable		Sample
BMI (kg/m^2^)	Median [Q1; Q3]	23.3 [20.6; 26.2]
	Minimum	14.1
	Maximum	36.7
Dialysis duration (months)	Median [Q1; Q3]	58 [22; 108]
	Minimum	3
	Maximum	371
Dialysis frequency, *n* (%)	3 times per week	324 (90.3)
	Non-3 times per week	35 (9.7)
Kt/V	Median [Q1; Q3]	1.3 [1.2; 1.6]
	Minimum	0.5
	Maximum	5.3
URR	Median [Q1; Q3]	67.2 [62.3; 72.3]
	Minimum	40.8
	Maximum	96.2
Dialysis modality, *n* (%)	High-flux	165 (46.0)
	Conventional	194 (54.0)
Primary disease, *n* (%)	Chronic glomerulonephritis	106 (29.5)
	Diabetic nephropathy	100 (27.9)
	Hypertensive kidney damage	84 (23.4)
	Other	69 (19.2)
Comorbidities, *n* (%)	Hypertension	182 (50.7)
	Hyperlipidemia	52 (14.5)
	Ischemic heart disease	57 (15.9)
	Diabetes	68 (18.9)
	Other	27 (7.5)
	None	102 (28.4)
Complications, *n* (%)	Renal anemia	266 (74.1)
	Chronic Kidney Disease-mineral and bone disorder	271 (75.5)
	Renal hypertension	151 (42.1)
	Malnutrition	45 (12.5)
	Dialysis hypotension	45 (12.5)
	Hyperuricemia	44 (12.3)
	Other	20 (5.6)
Polypharmacy*, *n* (%)	None	70 (19.5)
	Polypharmacy	195 (54.3)
	Excessive polypharmacy	94 (26.2)

Q1, First quartile; Q3, Third quartile; BMI, Body Mass Index; URR, Urea Reduction Ratio; Polypharmacy classification based on the number of medications used by the patient. “None” refers to 1–4 types of medications; “Polypharmacy” refers to 5–9 types; “Excessive polypharmacy” refers to 10 or more types of medications.

### 3.2 DSI results

This study used the DSI to assess symptom distress in 359 patients. The median number of symptoms reported by the sample was 13, and the median total DSI score was 24 (**Table 3**). The frequency of patient responses to each symptom in the DSI is detailed in **Table 4**.

**Table 3:** Overview of symptom distress in patients.

Indicator	Median [Q1; Q3]	Minimum	Maximum
Number of symptoms (items)	13 [9; 17]	2	27
Total score (points)	24 [18; 32]	6	77

Q1, First quartile; Q3, Third quartile.

**Table 4:** Frequency of patient responses to DSI (N (%)).

DSI					
Symptom	0	1	2	3	4
Constipation	241 (67.1)	65 (18.1)	33 (9.2)	20 (5.6)	0 (0)
Nausea	202 (56.3)	103 (28.7)	31 (8.6)	11 (3.1)	12 (3.3)
Vomiting	342 (95.3)	17 (4.7)	0 (0)	0 (0)	0 (0)
Diarrhea	284 (79.1)	55 (15.3)	10 (2.8)	10 (2.8)	0 (0)
Decreased appetite	204 (56.8)	100 (27.9)	30 (8.4)	13 (3.6)	12 (3.3)
Muscle cramps	175 (48.7)	124 (34.5)	37 (10.3)	14 (3.9)	9 (2.5)
Swelling in legs	279 (77.7)	50 (13.9)	28 (7.8)	2 (0.6)	0 (0)
Shortness of breath	230 (64.1)	83 (23.1)	27 (7.5)	11 (3.1)	8 (2.2)
Lightheadedness or dizziness	188 (52.4)	114 (31.8)	42 (11.7)	6 (1.7)	9 (2.5)
Restless legs or difficulty keeping legs still	175 (48.7)	124 (34.5)	37 (10.3)	14 (3.9)	9 (2.5)
Numbness or tingling in feet	209 (58.2)	100 (27.9)	33 (9.2)	10 (2.8)	7 (1.9)
Feeling tired or lack of energy	121 (33.7)	165 (46.0)	50 (13.9)	10 (2.8)	13 (3.6)
Cough	305 (85.0)	38 (10.6)	15 (4.2)	1 (0.3)	0 (0)
Dry mouth	129 (35.9)	158 (44.0)	58 (16.2)	14 (3.9)	0 (0)
Bone or joint pain	310 (86.4)	18 (5.0)	24 (6.7)	7 (1.9)	0 (0)
Chest pain	272 (75.8)	64 (17.8)	17 (4.7)	5 (1.4)	1 (0.3)
Headache	326 (90.8)	21 (5.8)	11 (3.1)	1 (0.3)	0 (0)
Muscle soreness	219 (61.0)	91 (25.3)	33 (9.2)	10 (2.8)	6 (1.7)
Difficulty concentrating	138 (38.4)	128 (35.7)	56 (15.6)	17 (4.7)	20 (5.6)
Dry skin	109 (30.4)	161 (44.8)	62 (17.3)	13 (3.6)	14 (3.9)
Itching	112 (31.2)	147 (40.9)	59 (16.4)	19 (5.3)	22 (6.1)
Worrying*	1 (0.3)	135 (37.6)	118 (32.9)	74 (20.6)	18 (5.0)
Feeling nervous	354 (98.6)	3 (0.8)	2 (0.6)	0 (0)	0 (0)
Trouble falling asleep	33 (9.2)	63 (17.5)	73 (20.3)	46 (12.8)	144 (40.1)
Trouble staying asleep	75 (20.9)	134 (37.3)	56 (15.6)	36 (10.0)	58 (16.2)
Feeling irritable	158 (44.0)	145 (40.4)	39 (10.9)	4 (1.1)	13 (3.6)
Feeling sad	342 (95.3)	7 (1.9)	8 (2.2)	2 (0.6)	0 (0)
Feeling anxious	345 (96.1)	6 (1.7)	7 (1.9)	1 (0.3)	0 (0)
Decreased interest in sex	29 (8.1)	16 (4.5)	16 (4.5)	2 (0.6)	296 (82.5)
Difficulty becoming sexually aroused	31 (8.6)	17 (4.7)	13 (3.6)	2 (0.6)	296 (82.5)

DSI, Dialysis Symptom Index; *1 missing; 0, Not bothersome at all; 1, A little bit bothersome; 2, Somewhat bothersome; 3, Quite bothersome; 4, Very bothersome.

### 3.3 KDQOL-SF™ 1.3 results

This study used the KDQOL-SF™ 1.3 scale to assess the quality of life of patients. The scale includes kidney disease-targeted dimensions and the SF-36 generic core dimensions. The patients’ scores in each domain are detailed in **Table 5**.

**Table 5:** Scores of each domain in the KDQOL-SF™ 1.3 scale for CKD Stage 5 hemodialysis patients.

Domain	Score (Median [Q1; Q3])
Symptom/Problem List	86.5 [75; 93.2]
Effects of Kidney Disease	68.8 [50; 81.3]
Burden of Kidney Disease	50 [25; 62.5]
Work Status	50 [0; 50]
Cognitive Function	86.7 [73.3; 100]
Quality of Social Interaction	86.7 [66.7; 93.3]
Sexual Function #	87.5 [65.6; 100]
Sleep	62.5 [50; 80]
Social Support	83.3 [66.7; 100]
Dialysis Staff Encouragement	100 [100; 100]
Patient Satisfaction	83.3 [83.3; 100]
Physical Functioning	75 [55; 90]
Role Limitations - Physical	50 [0; 100]
Role Limitations - Emotional	100 [0; 100]
Social Functioning	75 [50; 87.5]
Emotional Well-Being	76 [60; 88]
Pain	90 [77.5; 100]
Energy/Fatigue	70 [55; 85]
General Health Perceptions	45 [35; 65]
Overall Health Rating	60 [50; 80]
Change in Health	50 [50; 75]

Q1, First quartile; Q3, Third quartile. The “Sexual Function” domain was scored only for patients reporting sexual activity (range 0-100, with 0 indicating severe dysfunction). Patients with no sexual activity were treated as missing values and excluded from the statistics.

In the kidney disease-targeted dimensions, the highest score was in the “Dialysis Staff Encouragement” domain, with a median of 100. The scores in the “Symptom/Problem List” (86.5), “Cognitive Function” (86.7), and “Quality of Social Interaction” (86.7) domains were also relatively high, indicating better quality of life in these areas. In contrast, the patients scored lower in the “Burden of Kidney Disease” (50), “Work Status” (50), and “Sleep” (62.5) domains.

In the SF-36 generic core dimensions, the highest score was in the “Role Limitations - Emotional” domain, with a median of 100, followed by “Pain” (90). In contrast, the scores in the “General Health Perceptions” (45) and “Role Limitations - Physical” (50) domains were relatively low.

### 3.4 Univariate analysis of total DSI score and patient characteristics

Gender: The median total DSI score for males was 24.0 (17.5, 31), and for females, it was 24.5 (18, 32). The overall distribution of DSI total scores between the two groups showed no statistically significant difference (*Z* = 1.173, *P* = 0.241).

Age: The results showed that there was no statistically significant association between the total DSI score and age (*r* = 0.329, *P* < 0.001).

Education level: The median total DSI score was 32 (22.5, 36.3) in the primary school or below group, 24.5 (19, 32.8) in the junior high school/senior high school/technical secondary school/vocational high school group, and 23 (16, 29) in the junior college/bachelor’s degree or above group. There was a statistically significant difference in the overall distribution of total DSI scores among the three groups (*H* = 8.831, *P* = 0.012). *Post hoc* comparisons showed a statistically significant difference between the primary school or below group and the junior college/bachelor’s degree or above group (*P* = 0.017). However, no statistically significant differences were found between the primary school or below group and the junior high school/senior high school/technical secondary school/vocational high school group, nor between the junior high school/senior high school/technical secondary school/vocational high school group and the junior college/bachelor’s degree or above group (*P* > 0.05).

Marital status: The median total DSI score was 24 (18, 32) in the married group and 22 (17, 30.3) in the unmarried group. There was no statistically significant difference in the overall distribution of total DSI scores between the two groups (*Z* = 0.830, *P* = 0.406).

Employment status: The median total DSI score was 22 (16, 28) in the employed group and 25.5 (20, 34) in the unemployed group. There was a statistically significant difference in the overall distribution of total DSI scores between the two groups (*Z* = 3.390, *P* < 0.001).

BMI: The results showed that there was no statistically significant association between the total DSI score and BMI (*r* = -0.011, *P* = 0.830).

Dialysis vintage: The results showed that there was no statistically significant association between the total DSI score and dialysis vintage (*r* = 0.015, *P* = 0.772).

Dialysis frequency: The median total DSI score was 25 (19, 32) in the 3-times-per-week group and 20 (15, 27) in the non–3-times-per-week group. There was a statistically significant difference in the overall distribution of total DSI scores between the two groups (*Z* = 3.258, *P* = 0.001).

Kt/V: The results showed that there was no statistically significant association between the total DSI score and Kt/V (*r* = 0.018, *P* = 0.732).

URR: The results showed that there was no statistically significant association between the total DSI score and URR (*r* = 0.042, *P* = 0.423).

Dialysis modality: The median total DSI score was 28 (21, 35) in the high-flux group and 21 (17, 28) in the conventional dialysis group. There was a statistically significant difference in the overall distribution of total DSI scores between the two groups (*Z* = 5.444, *P* < 0.001).

Primary disease: The median total DSI score was 23 (16.8, 31.3) in the chronic glomerulonephritis group, 27 (20.3, 35) in the diabetic nephropathy group, 21 (17, 29) in the hypertensive nephropathy group, and 25 (21, 30.5) in the other causes group. There was a statistically significant difference in the overall distribution of total DSI scores among the four groups (*H* = 10.357, *P* = 0.016).

*Post hoc* comparisons showed a statistically significant difference between the diabetic nephropathy group and the hypertensive nephropathy group (*P* = 0.027). However, no statistically significant differences were found between the hypertensive nephropathy group and the chronic glomerulonephritis group, between the hypertensive nephropathy group and the other causes group, between the chronic glomerulonephritis group and the other causes group, between the chronic glomerulonephritis group and the diabetic nephropathy group, or between the other causes group and the diabetic nephropathy group (*P* > 0.05).

Comorbidities: (1) Hypertension: The median total DSI score was 25 (19, 33) in patients with hypertension and 24 (17.5, 30) in those without hypertension. There was no statistically significant difference in the overall distribution of total DSI scores between the two groups (*Z* = 1.384, *P* = 0.166).

(2) Hyperlipidemia: The median total DSI score was 29.5 (23, 35.8) in patients with hyperlipidemia and 24 (17, 31) in those without hyperlipidemia. There was a statistically significant difference in the overall distribution of total DSI scores between the two groups (*Z* = 3.531, *P* < 0.001).

3. Ischemic heart disease: The median total DSI score was 28 (23, 34.5) in patients with ischemic heart disease and 23 (17, 31) in those without ischemic heart disease. There was a statistically significant difference in the overall distribution of total DSI scores between the two groups (*Z* = 3.142, *P* = 0.002).

4. Diabetes mellitus: The median total DSI score was 26.5 (20.3, 34.8) in patients with diabetes mellitus and 24 (17, 31) in those without diabetes mellitus. There was a statistically significant difference in the overall distribution of total DSI scores between the two groups (*Z* = 2.532, *P* = 0.011).

5. Other comorbidities: The median total DSI score was 25 (19, 32) in patients with other comorbidities and 24 (18, 31.8) in those without other comorbidities. There was no statistically significant difference in the overall distribution of total DSI scores between the two groups (*Z* = 0.872, *P* = 0.383).

6. Presence of comorbidities: The median total DSI score was 25 (20, 33) in patients with comorbidities and 21 (15.8, 28) in those without comorbidities. There was a statistically significant difference in the overall distribution of total DSI scores between the two groups (*Z* = 4.094, *P* < 0.001).

Complications: (1) Renal anemia: The median total DSI score was 25 (20, 33) in patients with renal anemia and 20 (14.5, 28) in those without renal anemia. There was a statistically significant difference in the overall distribution of total DSI scores between the two groups (*Z* = 4.461, *P* < 0.001).

2. Chronic Kidney Disease–mineral and bone disorder (CKD-MBD): The median total DSI score was 25 (19, 32) in patients with CKD-MBD and 22 (17.3, 31) in those without CKD-MBD. There was no statistically significant difference in the overall distribution of total DSI scores between the two groups (*Z* = 1.389, *P* = 0.165).

3. Renal hypertension: The median total DSI score was 24 (19, 33) in patients with renal hypertension and 24 (17, 31) in those without renal hypertension. There was no statistically significant difference in the overall distribution of total DSI scores between the two groups (*Z* = 0.958, *P* = 0.338).

4. Malnutrition: The median total DSI score was 26 (19.5, 32.5) in patients with malnutrition and 24 (18, 31.3) in those without malnutrition. There was no statistically significant difference in the overall distribution of total DSI scores between the two groups (*Z* = 0.752, *P* = 0.452).

5. Intradialytic hypotension: The median total DSI score was 23 (18.5, 31) in patients with intradialytic hypotension and 24 (18, 32) in those without intradialytic hypotension. There was no statistically significant difference in the overall distribution of total DSI scores between the two groups (*Z* = 0.367, *P* = 0.714).

6. Hyperuricemia: The median total DSI score was 27.5 (21.3, 31) in patients with hyperuricemia and 24 (18, 32) in those without hyperuricemia. There was no statistically significant difference in the overall distribution of total DSI scores between the two groups (*Z* = 1.847, *P* = 0.065).

7. Other complications: The median total DSI score was 26.5 (23.3, 31.3) in patients with other complications and 24 (18, 32) in those without other complications. There was no statistically significant difference in the overall distribution of total DSI scores between the two groups (*Z* = 1.467, *P* = 0.142).

Polypharmacy status: The median total DSI score was 24 (19, 32.5) in the non-polypharmacy group, 22 (16, 28) in the polypharmacy group, and 29 (23, 34.3) in the excessive polypharmacy group. There was a statistically significant difference in the overall distribution of total DSI scores among the three groups (*H* = 20.572, *P* < 0.001). *Post hoc* comparisons showed that the total DSI score in the excessive polypharmacy group was significantly higher than that in both the polypharmacy group (*P* < 0.001) and the non-polypharmacy group (*P* = 0.039). However, no statistically significant difference was observed between the polypharmacy group and the non-polypharmacy group (*P* > 0.05). See **Table 6** for details.

**Table 6:** Univariate analysis of the total DSI score.

Variable	Group	*n* (%)	Total DSI score (Median [Q1, Q3])	Effect size	*P* value
Sociodemographic characteristics					
Sex	Male	201 (56.0)	24.0 [17.5; 31]	*Z* = 1.173	0.241
	Female	158 (44.0)	24.5 [18; 32]		
Age	Continuous variables			*r* = 0.329	<0.001
Education level	Primary school or below	18 (5.0)	32 [22.5; 36.3]*	*H* = 8.831	0.012
	Junior high school/Senior high school/Technical secondary school/Vocational high school	224 (62.4)	24.5 [19; 32.8]		
	Junior college/Bachelor’s degree or above	117 (32.6)	23 [16; 29]		
Marital status	Married	309 (86.1)	24 [18; 32]	*Z* = 0.830	0.406
	Unmarried	50 (13.9)	22 [17; 30.3]		
Employment status	Employed	155 (43.2)	22 [16; 28]	*Z* = 3.390	<0.001
	Unemployed	200 (55.7)	25.5 [20; 34]		
Disease-related characteristics					
BMI	Continuous variables			*r* = -0.011	0.830
Dialysis vintage	Continuous variables			*r* = 0.015	0.772
Dialysis frequency	3 times per week	324 (90.3)	25 [19; 32]	*Z* = 3.258	0.001
	non–3-times-per-week	35 (9.7)	20 [15; 27]		
Kt/V	Continuous variables			*r* = 0.018	0.732
URR	Continuous variables			*r* = 0.042	0.423
Dialysis modality	High-flux	165 (46.0)	28 [21; 35]	*Z* = 5.444	<0.001
	Conventional	194 (54.0)	21 [17; 28]		
Primary disease	Chronic glomerulonephritis	106 (29.5)	23 [16.8; 31.3]	*H* = 10.357	0.016
	Diabetic nephropathy	100 (27.9)	27 [20.3; 35] #		
	Hypertensive nephropathy	84 (23.4)	21 [17; 29]		
	Other causes	69 (19.2)	25 [21; 30.5]		
Comorbidities					
Hypertension	Yes	182 (50.7)	25 [19; 33]	*Z* = 1.384	0.166
	No	177 (49.3)	24 [17.5; 30]		
Hyperlipidemia	Yes	52 (14.5)	29.5 [23; 35.8]	*Z* = 3.531	<0.001
	No	307 (85.5)	24 [17; 31]		
Ischemic heart disease	Yes	57 (15.9)	28 [23; 34.5]	*Z* = 3.142	0.002
	No	302 (84.1)	23 [17; 31]		
Diabetes mellitus	Yes	68 (18.9)	26.5 [20.3; 34.8]	*Z* = 2.532	0.011
	No	291 (81.1)	24 [17; 31]		
Other comorbidities	Yes	27 (7.5)	25 [19; 32]	*Z* = 0.872	0.383
	No	332 (92.5)	24 [18; 31.8]		
Presence of comorbidities	Yes	257 (71.6)	25 [20; 33]	*Z* = 4.094	<0.001
	No	102 (28.4)	21 [15.8; 28]		
Complications					
Renal anemia	Yes	266 (74.1)	25 [20; 33]	*Z* = 4.461	<0.001
	No	93 (25.9)	20 [14.5; 28]		
CKD-MBD	Yes	271 (75.5)	25 [19; 32]	*Z* = 1.389	0.165
	No	88 (24.5)	22 [17.3; 31]		
Renal hypertension	Yes	151 (42.1)	24 [19; 33]	*Z* = 0.958	0.338
	No	208 (57.9)	24 [17; 31]		
Malnutrition	Yes	45 (12.5)	26 [19.5; 32.5]	*Z* = 0.752	0.452
	No	314 (87.5)	24 [18; 31.3]		
Intradialytic hypotension	Yes	45 (12.5)	23 [18.5; 31]	*Z* = 0.367	0.714
	No	314 (87.5)	24 [18; 32]		
Hyperuricemia	Yes	44 (12.3)	27.5 [21.3; 31]	*Z* = 1.847	0.065
	No	315 (87.7)	24 [18; 32]		
Other complications	Yes	16 (4.5)	26.5 [23.3; 31.3]	*Z* = 1.467	0.142
	No	343 (95.5)	24 [18; 32]		
Polypharmacy status	Non-polypharmacy	70 (19.5)	24 [19; 32.5]	*H* = 20.572	<0.001
	Polypharmacy	195 (54.3)	22 [16; 28]		
	Excessive polypharmacy	94 (26.2)	29 [23; 34.3]**		

DSI, Dialysis Symptom Index; BMI, Body Mass Index; URR, Urea Reduction Ratio; CKD-MBD, Chronic Kidney Disease-Mineral and Bone Disorder. *Compared with the junior college/bachelor’s degree or above group, *P* < 0.05. # Compared with the hypertensive nephropathy group, *P* < 0.05. **Compared with the polypharmacy group and the non-polypharmacy group, both *P* < 0.05.

### 3.5 Correlation analysis between the DSI and KDQOL-SF™ 1.3

Symptom/Problem list: The results showed that there was a statistically significant association between the total DSI score and the Symptom/Problem list domain (*r* = -0.890, *P* < 0.001).

Effects of kidney disease: The results showed that there was a statistically significant association between the total DSI score and the Effects of kidney disease domain (*r* = -0.693, *P* < 0.001).

Burden of kidney disease: The results showed that there was a statistically significant association between the total DSI score and the Burden of kidney disease domain (*r* = -0.458, *P* < 0.001).

Work status: The results showed that there was a statistically significant association between the total DSI score and the Work status domain (*r* = -0.235, *P* < 0.001).

Cognitive function: The results showed that there was a statistically significant association between the total DSI score and the Cognitive function domain (*r* = -0.578, *P* < 0.001).

Quality of social interaction: The results showed that there was a statistically significant association between the total DSI score and the Quality of social interaction domain (*r* = -0.471, *P* < 0.001).

Sexual function: The results showed that there was a statistically significant association between the total DSI score and the Sexual function domain (*r* = -0.548, *P* < 0.001).

Sleep: The results showed that there was a statistically significant association between the total DSI score and the Sleep domain (*r* = -0.492, *P* < 0.001).

Social support: The results showed that there was a statistically significant association between the total DSI score and the Social support domain (*r* = -0.323, *P* < 0.001).

Dialysis staff encouragement: The results showed that there was a statistically significant association between the total DSI score and the Dialysis staff encouragement domain (*r* = -0.177, *P* < 0.001).

Patient satisfaction: The results showed that there was a statistically significant association between the total DSI score and the Patient satisfaction domain (*r* = -0.131, *P* = 0.013).

Physical functioning: The results showed that there was a statistically significant association between the total DSI score and the Physical functioning domain (*r* = -0.559, *P* < 0.001).

Role limitations - physical: The results showed that there was a statistically significant association between the total DSI score and the Role limitations - physical domain (*r* = -0.484, *P* < 0.001).

Role limitations - emotional: The results showed that there was a statistically significant association between the total DSI score and the Role limitations - emotional domain (*r* = -0.463, *P* < 0.001).

Social functioning: The results showed that there was a statistically significant association between the total DSI score and the Social functioning domain (*r* = -0.522, *P* < 0.001).

Emotional well-being: The results showed that there was a statistically significant association between the total DSI score and the Emotional well-being domain (*r* = -0.467, *P* < 0.001).

Pain: The results showed that there was a statistically significant association between the total DSI score and the Pain domain (*r* = -0.578, *P* < 0.001).

Energy/Fatigue: The results showed that there was a statistically significant association between the total DSI score and the Energy/Fatigue domain (*r* = -0.594, *P* < 0.001).

General health perceptions: The results showed that there was a statistically significant association between the total DSI score and the General health perceptions domain (*r* = -0.516, *P* < 0.001).

Overall health rating: The results showed that there was a statistically significant association between the total DSI score and the Overall health rating domain (*r* = -0.367, *P* < 0.001).

Change in health: The results showed that there was a statistically significant association between the total DSI score and the Change in health domain (*r* = -0.234, *P* < 0.001). See **Table 7** for details.

**Table 7:** Spearman correlation coefficients between the total DSI score and the domain scores of the KDQOL-SF™ 1.3.

Domain	*r*	*P* value
Symptom/Problem List	-0.890	<0.001
Effects of Kidney Disease	-0.693	<0.001
Burden of Kidney Disease	-0.458	<0.001
Work Status	-0.235	<0.001
Cognitive Function	-0.578	<0.001
Quality of Social Interaction	-0.471	<0.001
Sexual Function	-0.548	<0.001
Sleep	-0.492	<0.001
Social Support	-0.323	<0.001
Dialysis Staff Encouragement	-0.177	<0.001
Patient Satisfaction	-0.131	0.013
Physical Functioning	-0.559	<0.001
Role Limitations - Physical	-0.484	<0.001
Role Limitations - Emotional	-0.463	<0.001
Social Functioning	-0.522	<0.001
Emotional Well-Being	-0.467	<0.001
Pain	-0.578	<0.001
Energy/Fatigue	-0.594	<0.001
General Health Perceptions	-0.516	<0.001
Overall Health Rating	-0.367	<0.001
Change in Health	-0.234	<0.001

### 3.6 Multiple linear regression analysis of the total DSI score

The total DSI score was entered as the dependent variable, and sex, age, education level, marital status, employment status, BMI, dialysis vintage, dialysis frequency, Kt/V, URR, dialysis modality, primary disease, comorbidities, presence of comorbidities, complications, and polypharmacy status were included as independent variables in the multiple linear regression analysis.

The independent variables were coded as follows: sex (0 = male, 1 = female); education level (primary school or below as the reference category); marital status (0 = married, 1 = unmarried); employment status (0 = employed, 1 = unemployed); dialysis frequency (0 = 3 times per week, 1 = non–3-times-per-week); dialysis modality (0 = high-flux, 1 = conventional); primary disease (chronic glomerulonephritis as the reference category); comorbidities (hypertension as the reference category); presence of comorbidities (0 = yes, 1 = no); complications (renal anemia as the reference category); and polypharmacy status (non-polypharmacy as the reference category). Age, BMI, dialysis vintage, Kt/V, and URR were entered as continuous variables.

The results of the regression analysis (**Table 8**) showed that the adjusted R^2^ of the model was 0.191, indicating that the included independent variables explained 19.1% of the variance in the total DSI score. The Durbin–Watson statistic was 1.894, suggesting independence of residuals. Collinearity diagnostics showed that the variance inflation factors (VIF) for all variables were less than 10, indicating no serious multicollinearity among the independent variables.

**Table 8:** Multiple linear regression analysis of the total DSI score.

Coefficients^a^							
Model	Unstandardized coefficients		Standardized coefficients	t	Sig.	Collinearity statistics	
	B	Std. error	Beta			Tolerance	VIF
(Constant)	34.283	11.089		3.092	0.002		
Sex							
Female	1.331	1.198	0.062	1.111	0.267	0.727	1.375
Male	0						
Age	0.209	0.054	0.240	3.895	<0.001	0.598	1.673
Education level							
Junior high school/Senior high school/Technical secondary school/Vocational high school	-4.860	2.450	-0.221	-1.983	0.048	0.182	5.482
Junior college/Bachelor’s degree or above	-5.880	2.598	-0.258	-2.263	0.024	0.173	5.772
Primary school or below	0						
Marital status							
Unmarried	1.483	1.598	0.048	0.928	0.354	0.839	1.192
Married	0						
Employment status							
Unemployed	-0.012	1.221	-0.001	-0.010	0.992	0.702	1.424
Employed	0						
BMI	-0.054	0.148	-0.021	-0.362	0.718	0.702	1.424
Dialysis vintage	0.027	0.008	0.187	3.242	0.001	0.679	1.473
Dialysis frequency							
Non–3-times-per-week	-3.315	1.816	-0.092	-1.825	0.069	0.885	1.130
3 times per week	0						
Kt/V	1.415	2.464	0.054	0.574	0.566	0.252	3.971
URR	-0.207	0.148	-0.150	-1.398	0.163	0.197	5.088
Dialysis modality							
Conventional	-5.453	1.352	-0.255	-4.034	<0.001	0.566	1.766
High-flux	0						
Primary disease							
Diabetic nephropathy	4.459	1.570	0.187	2.840	0.005	0.519	1.928
Hypertensive nephropathy	1.957	1.528	0.078	1.281	0.201	0.614	1.628
Other causes	3.097	1.591	0.114	1.946	0.052	0.654	1.530
Chronic glomerulonephritis	0						
Comorbidities							
Hyperlipidemia	-1.563	1.773	-0.052	-0.881	0.379	0.660	1.516
Ischemic heart disease	0.220	1.619	0.008	0.136	0.892	0.734	1.362
Diabetes mellitus	-2.205	1.520	-0.081	-1.451	0.148	0.725	1.380
Other comorbidities	-1.069	2.151	-0.026	-0.497	0.620	0.798	1.253
Hypertension	0						
Presence of comorbidities							
No	-0.051	1.483	-0.002	-0.034	0.973	0.575	1.740
Yes	0						
Complications							
CKD-MBD	0.944	1.443	0.038	0.654	0.514	0.667	1.500
Renal hypertension	-2.023	1.249	-0.094	-1.619	0.106	0.676	1.480
Malnutrition	-0.503	1.622	-0.016	-0.310	0.757	0.890	1.123
Intradialytic hypotension	0.132	1.819	0.004	0.072	0.942	0.709	1.411
Hyperuricemia	-0.055	1.882	-0.002	-0.029	0.977	0.675	1.482
Other complications	0.773	2.739	0.015	0.282	0.778	0.804	1.243
Renal anemia	0						
Polypharmacy status							
Polypharmacy	-1.121	1.439	-0.052	-0.779	0.436	0.500	1.999
Excessive polypharmacy	1.540	1.835	0.064	0.839	0.402	0.395	2.532
Non-polypharmacy	0						

^a^Dependent variable: Total DSI score. VIF, Variance Inflation Factor; DSI, Dialysis Symptom Index; BMI, Body Mass Index; URR, Urea Reduction Ratio; CKD-MBD, Chronic Kidney Disease-Mineral and Bone Disorder.

After controlling for other variables, age (B = 0.209, β = 0.240, *t* = 3.895, *P* < 0.001), dialysis vintage (B = 0.027, β = 0.187, *t* = 3.242, *P* = 0.001), conventional dialysis modality (B = -5.453, β = -0.255, *t* = -4.034, *P* < 0.001), and diabetic nephropathy as the primary disease (B = 4.459, β = 0.187, *t* = 2.840, *P* = 0.005) were significantly associated with the total DSI score.

Compared with patients with primary school education or below, those with junior high school/senior high school/technical secondary school/vocational high school education (B = -4.860, β = -0.221, *t* = -1.983, *P* = 0.048) and those with junior college/bachelor’s degree or above (B = –5.880, β = -0.258, *t* = -2.263, *P* = 0.024) had lower total DSI scores.

The remaining independent variables (sex, marital status, employment status, BMI, dialysis frequency, Kt/V, URR, comorbidities, presence of comorbidities, complications, and polypharmacy status) were not significantly associated with the total DSI score (*P* > 0.05).

### 3.7 Multiple linear regression analysis of the association between symptom distress and quality of life

To examine the independent association between symptom distress (total DSI score) and each domain of quality of life among patients with CKD Stage 5 undergoing hemodialysis, multiple linear regression analyses were performed with the domain scores of the KDQOL-SF™ 1.3 as dependent variables. In each regression model, the total DSI score was entered as the primary independent variable, while sociodemographic characteristics (sex, age, education level, marital status, and employment status) and disease-related variables (BMI, dialysis vintage, dialysis frequency, Kt/V, URR, dialysis modality, primary disease, comorbidities, complications, and polypharmacy status) were included as covariates. The coding of independent variables was consistent with that described above.

The R^2^ values of the models ranged from 0.139 to 0.824, indicating varying explanatory power across different quality-of-life domains. The models showed particularly strong explanatory power for the Symptoms/Problems list (R^2^ = 0.824) and Sexual Function (R^2^ = 0.688) domains. The Durbin–Watson statistics in all models were close to 2, suggesting good independence of residuals. Detailed results are presented in **Supplementary Table S1**.

The main results showed that, after controlling for all potential confounding factors, the total DSI score (severity of symptom distress) was an independent predictor of multiple domains of quality of life in these patients.

1. Association with kidney disease–targeted domains: The total DSI score was significantly negatively associated with all Kidney Disease-Targeted Dimensions except Patient Satisfaction. Specifically, higher total DSI scores (indicating more severe symptom distress) were associated with lower scores in the Symptom/Problem List (B = -1.197, *P* < 0.001), Effects of Kidney Disease (B = -1.328, *P* < 0.001), Burden of Kidney Disease (B = -1.080, *P* < 0.001), Work Status (B = -0.534, *P* = 0.004), Cognitive Function (B = -1.093, *P* < 0.001), Quality of Social Interaction (B = -0.878, *P* < 0.001), Sexual Function (B = -1.834, *P* = 0.002), Sleep (B = -0.799, *P* < 0.001), Social Support (B = -0.433, *P* < 0.001), and Dialysis Staff Encouragement (B = -0.225, *P* = 0.009), indicating more severe impairment in quality of life.
2. Association with the SF-36 Generic Core Dimensions: The total DSI score was significantly negatively associated with all SF-36 generic core domains. Specifically, higher total DSI scores were significantly associated with lower scores in Physical Functioning (B = -1.008, *P* < 0.001), Role Limitations - Physical (B = -1.601, *P* < 0.001), Role Limitations - Emotional (B = -1.911, *P* < 0.001), Social Functioning (B = -1.272, *P* < 0.001), Emotional Well-Being (B = -0.853, *P* < 0.001), Pain (B = -1.173, *P* < 0.001), Energy/Fatigue (B = -1.044, *P* < 0.001), General Health Perceptions (B = -0.880, *P* < 0.001), Overall Health Rating (B = -0.722, *P* < 0.001), and Change in Health (B = -0.730, *P* < 0.001).

These findings indicate that patients with more severe symptom distress have poorer quality of life across physical, psychological, and social functioning domains.

In addition, several covariates showed independent associations with specific quality-of-life domains.

1. Age: Increasing age was significantly associated with higher scores in Cognitive Function (B = 0.191, *P* = 0.023) and Quality of Social Interaction (B = 0.211, *P* = 0.013), but was significantly associated with lower scores in Physical Functioning (B = -0.543, *P* < 0.001), Role Limitations - Physical (B = -0.732, *P* < 0.001), and Role Limitations - Emotional (B = -0.535, *P* = 0.012).
2. Dialysis modality: Compared with high-flux dialysis, conventional dialysis was significantly associated with poorer Work Status (B = 15.204, *P* = 0.001), Quality of Social Interaction (B = 9.752, *P* < 0.001), Dialysis Staff Encouragement (B = 8.777, *P* < 0.001), Patient Satisfaction (B = 9.830, *P* < 0.001), and Role Limitations - Emotional (B = 11.584, *P* = 0.030). At the same time, conventional dialysis was also significantly associated with lower Change in Health scores (B = -11.877, *P* = 0.002).
3. Primary disease: Using chronic glomerulonephritis as the reference category, patients with diabetic nephropathy had significantly higher scores in Cognitive Function (B = 7.589, *P* = 0.002) but significantly lower scores in Physical Functioning (B = -12.356, *P* < 0.001). Patients with hypertensive nephropathy had significantly higher scores in the Burden of Kidney Disease (B = 10.558, *P* = 0.002) and General Health Perceptions (B = 6.803, *P* = 0.012) domains.
4. BMI: Higher BMI was significantly associated with poorer Physical Functioning (B = -0.920, *P* = 0.002) and more severe Pain (B = -0.958, *P* < 0.001).
5. Dialysis vintage: Longer dialysis vintage was significantly associated with higher scores in the Symptom/Problem List domain (B = -0.013, *P* = 0.019), but was also significantly associated with lower scores in the Pain (B = -0.042, *P* = 0.004) and Change in Health (B = -0.109, *P* < 0.001) domains.
6. Complications: Patients with renal hypertension had significantly poorer scores in Sleep (B = 5.082, *P* = 0.025), Social Support (B = 7.781, *P* = 0.008), and Energy/Fatigue (B = 4.658, *P* = 0.028). In contrast, patients with CKD-MBD had significantly better scores in Physical Functioning (B = 8.430, *P* = 0.004).
7. Presence of comorbidities: Compared with patients with comorbidities, those without comorbidities had significantly higher scores in Emotional Well-Being (B = -8.593, *P* < 0.001) and Energy/Fatigue (B = -5.659, *P* = 0.024).

## 4 Discussion

### 4.1 Current status of symptom distress among patients with CKD Stage 5 undergoing hemodialysis

In this study, the DSI was used to systematically assess symptom distress in 359 patients with CKD Stage 5 undergoing hemodialysis. The results showed that patients reported an average of 13 symptoms, with a median total DSI score of 24. These findings are generally consistent with those reported by Georges et al. in Cameroon, where patients experienced a median of approximately 12 symptoms per individual, suggesting that the burden of symptoms is pervasive across different healthcare systems and cultural contexts (22).

Regarding symptom prevalence, sexual dysfunction (decreased interest in sex 91.9%, difficulty becoming sexually aroused 91.4%) and sleep disturbances (trouble falling asleep 90.8%, trouble staying asleep 79.1%) ranked highest. Compared with previous studies, the prevalence rates reported in the present study were relatively high. For example, Imhof et al. reported a combined prevalence of sexual dysfunction of 87% among female hemodialysis patients (23), and Tan et al. reported an insomnia prevalence of 68% (24). These findings may be attributed to multiple factors. From a physiological perspective, the median age of the patients in this study was 60 years, and aging itself is associated with a physiological decline in hormone levels. The accumulation of uremic toxins may lead to neuroendocrine disturbances, while endothelial dysfunction caused by vascular access and cardiovascular complications may further exacerbate erectile dysfunction (25). Sleep disturbances are widely recognized as one of the most common nonspecific symptoms in patients undergoing hemodialysis and are significantly associated with pruritus, pain, and uremic toxin accumulation (26). From a psychosocial perspective, long-term dialysis is often accompanied by changes in body image, loss of role function, uncertainty about the future, and related anxiety and depressive symptoms. These factors may not only aggravate decreased libido and arousal difficulties but also interfere with the initiation and maintenance of sleep by increasing psychological burden. In addition, cultural factors should not be overlooked. In the context of traditional Chinese culture, sexual topics are considered highly sensitive, which may influence patients’ willingness and accuracy in reporting related problems in questionnaire surveys. A qualitative study by An et al. on Chinese hemodialysis nurses also found that healthcare professionals themselves felt embarrassed when discussing sexual dysfunction and regarded sexuality as a “private topic” unsuitable for public discussion (27). Moreover, the high prevalence of emotional symptoms such as worrying (99.7%) may reflect patients’ profound concerns about disease prognosis and dependence in daily life.

It is noteworthy that the DSI indicated a prevalence of sexual dysfunction exceeding 91%, whereas the median score for the Sexual Function domain of the KDQOL-SF™ 1.3 reached 87.5. This discrepancy highlights the influence of measurement tools on the presentation of results. The Sexual Function domain of the KDQOL-SF™ 1.3 is scored only among patients who report being sexually active, with those reporting no sexual activity treated as missing values. Therefore, the relatively high score reflects only the subgroup of patients who remain sexually active, rather than the sexual function status of the entire study population. These findings suggest that, when interpreting symptom prevalence and severity, it is essential to consider differences in instrument design, the potential impact of cultural factors on willingness to report sensitive issues, and the underlying physiological and psychological mechanisms of the symptoms themselves.

The high prevalence of sexual dysfunction and sleep disturbances suggests that these symptoms may constitute key components of a core symptom cluster in patients with CKD Stage 5 undergoing hemodialysis. They are likely the result of the complex interplay among uremic toxin accumulation, chronic inflammation, endocrine dysregulation, and psychosocial factors. These findings indicate that clinical interventions should move beyond the management of isolated physiological symptoms and instead adopt integrated, multisymptom strategies that incorporate psychological support and rehabilitation of social functioning. Future research may benefit from combining multiple assessment instruments with qualitative interviews to more comprehensively capture patients’ lived experiences and to minimize potential measurement bias associated with reliance on a single tool.

It is noteworthy that several “classic” dialysis-related symptoms—such as dry skin (69.6%), itching (68.8%), and feeling tired or lack of energy (66.3%)—remain highly prevalent. Dry skin and itching may be associated with the accumulation of uremic toxins, impairment of the skin barrier function, and secondary hyperparathyroidism. Feeling tired or lack of energy, in contrast, may be attributable to multiple factors, including chronic inflammation, renal anemia, malnutrition, and prolonged post-dialysis recovery time. These findings suggest that optimal management of underlying complications—such as correcting calcium–phosphate metabolism disorders and improving anemia—remains a key strategy for effective symptom control in this population.

Emotional and cognitive symptoms should not be overlooked. The prevalence of worrying reached as high as 99.7%. Although most cases were of mild to moderate severity (with 70.5% scoring 1–2), its near universality suggests that psychological support should be incorporated into routine care. More than half of the patients also reported difficulty concentrating (61.6%) and feeling irritable (56.0%), reflecting the high burden of cognitive impairment and emotional instability in this population. These findings are consistent with those of Albuhayri et al., who likewise reported a high prevalence of psychological symptoms, including depression, among patients undergoing hemodialysis (28). Taken together, these results underscore the multidimensional nature of symptom distress in patients with CKD Stage 5 receiving hemodialysis and highlight the need for integrated interventions addressing physiological, psychological, and social domains.

### 4.2 Factors influencing the level of symptom distress in patients with CKD Stage 5 undergoing hemodialysis

Univariate analysis showed that age, education level, employment status, dialysis frequency, dialysis modality, primary disease, certain comorbidities (hyperlipidemia, ischemic heart disease, and diabetes mellitus), the presence of comorbidities, complications (renal anemia), and polypharmacy status were significantly associated with the total DSI score (*P* < 0.05). After further adjustment for potential confounding factors using multiple linear regression analysis, age, education level, dialysis vintage, dialysis modality, and primary disease (diabetic nephropathy) were identified as independent predictors of the total DSI score.

Age and dialysis vintage: Both age (B = 0.209, *P* < 0.001) and dialysis vintage (B = 0.027, *P* = 0.001) were independently and positively associated with the severity of symptom distress. Advancing age is accompanied by reduced physiological reserve, multimorbidity, and alterations in pharmacokinetics, which may exacerbate patients’ perception of symptoms and reduce their tolerance to symptom burden (8, 22, 29–33). A longer dialysis vintage implies prolonged exposure to uremic toxins and a higher risk of dialysis-related complications (e.g., CKD-MBD), thereby contributing to increased symptom distress.

Education level: Using primary school education or below as the reference category, patients with junior high school/senior high school/technical secondary school/vocational high school education (B = -4.860, *P* = 0.048) and those with junior college/bachelor’s degree or above (B = -5.880, *P* = 0.024) had significantly lower total DSI scores. This finding suggests a gradual decrease in symptom distress with increasing educational attainment. These results are consistent with the findings of Park et al. (34), indicating that individuals with higher education levels may possess greater health literacy. Such patients may be more proactive in seeking disease-related information, better able to understand medical instructions, and more capable of engaging in precise self-management (e.g., fluid control and medication adherence), thereby alleviating symptoms related to fluid overload and electrolyte imbalance. Clinically, greater attention should be directed toward symptom screening and health education among patients with lower educational levels.

Dialysis modality: Compared with high-flux dialysis, patients receiving conventional dialysis had significantly lower total DSI scores (B = -5.453, *P* < 0.001). High-flux dialysis is typically prescribed for patients with more severe clinical conditions, greater accumulation of middle and large molecular toxins, and a higher burden of complications. In other words, this association may be attributable to selection bias in clinical indications—patients with more severe symptom distress are preferentially assigned to more intensive dialysis regimens, rather than high-flux dialysis itself causing an increase in symptom burden. This also explains why, in the univariate analysis, the high-flux group showed higher total DSI scores, whereas in the multivariate regression analysis, conventional dialysis emerged as an independent factor associated with lower symptom distress.

Primary disease: Using chronic glomerulonephritis as the reference category, patients with diabetic nephropathy had significantly higher total DSI scores (B = 4.459, *P* = 0.005). As a systemic metabolic disorder, diabetes is associated with multiple complications—such as peripheral neuropathy, gastrointestinal dysmotility, and retinopathy—which may overlap with and exacerbate uremic symptoms. This interaction can result in a broader spectrum of manifestations, including limb pain, numbness, orthostatic hypotension, and gastrointestinal disturbances, thereby increasing overall symptom distress. These findings suggest that symptom management in patients with diabetic nephropathy should adopt a multidisciplinary, cross-specialty collaborative approach.

It is noteworthy that several variables that were statistically significant in the univariate analysis—including employment status, dialysis frequency, comorbidities (hyperlipidemia, ischemic heart disease, and diabetes mellitus), complications (renal anemia), and polypharmacy status—did not remain significant after inclusion in the multivariate regression model (*P* > 0.05). This finding suggests that the effects of these factors on symptom distress may be explained or attenuated by core variables such as age, dialysis vintage, education level, and primary disease type. In other words, advancing age, longer dialysis vintage, lower educational attainment, and diabetic nephropathy emerged as independent determinants of symptom distress severity, whereas the influence of other clinical characteristics did not retain statistical significance after adjustment for these key variables.

In addition, this study found no significant association between dialysis adequacy indicators (Kt/V and URR) and the total DSI score. This result suggests a potential dissociation between objectively measured solute clearance efficiency and patients’ subjectively reported symptom distress. Even when dialysis adequacy meets recommended clinical targets (with a median Kt/V of 1.3 and URR of 67.2% in this study), symptom distress may still arise from multiple factors beyond the accumulation of uremic toxins alone. One possible explanation is that traditional adequacy indicators, such as Kt/V and URR, primarily reflect the clearance of small-molecule solutes, whereas patients’ symptom experiences may be more strongly influenced by the accumulation of middle and large molecular toxins, chronic inflammatory states, and autonomic dysfunction. Therefore, relying solely on conventional dialysis adequacy parameters as indicators of treatment quality may not fully capture patients’ overall health status. Future evaluations of dialysis quality should ensure that objective adequacy targets are met while also integrating patient-reported outcomes, thereby shifting from a model centered on “adequacy indices” to one focused on “overall patient health”.

### 4.3 Current status of quality of life among patients with CKD Stage 5 undergoing hemodialysis

This study assessed patients’ quality of life using the KDQOL-SF™ 1.3 instrument. The results showed relatively low scores in several domains, including Work Status (median 50), Burden of Kidney Disease (median 50), Role Limitations - Physical (median 50), Change in Health (median 50), and General Health Perceptions (median 45). These findings are consistent with those reported by Vettath et al. (35) and De Pasquale et al. (36), suggesting that the disease exerts multidimensional negative effects on patients’ social functioning, psychological burden, and overall health evaluation. Specifically, the rigid time demands of dialysis treatment directly limit patients’ ability to engage in full-time employment. In addition, strict dietary restrictions, fluid control, and uncertainty about the future (e.g., waiting for kidney transplantation) impose substantial psychological stress, which may explain the low scores observed in the Burden of Kidney Disease domain.

In contrast, relatively high scores were observed in the domains of Dialysis Staff Encouragement (median 100), Role Limitations - Emotional (median 100), and Pain (median 90). These findings carry important clinical implications. First, the high score in the Pain domain suggests that current hemodialysis procedures and complication management strategies—such as appropriate anticoagulation and effective control of secondary hyperparathyroidism—may be relatively successful in alleviating physical discomfort. Second, the maximum score observed in the Dialysis Staff Encouragement domain underscores the unique value of healthcare professionals in long-term dialysis care. They serve not only as implementers of treatment protocols but also as crucial sources of emotional support for patients. Third, the high Role Limitations - Emotional score indicates that, despite the burden of chronic illness, patients are generally able to manage work and daily life responsibilities. This resilience may, in part, be attributable to the positive and sustained support provided by dialysis staff (37).

Overall, patients’ quality of life appears to demonstrate a pattern characterized by “marked physical functional limitation” coexisting with “relatively well-maintained psychological health.” This finding suggests that, although the disease itself and the dialysis treatment modality fundamentally restrict patients’ physical functioning, optimized medical management (e.g., effective pain control) and high-quality patient–provider interactions may play a crucial role in preserving psychological well-being and emotional functioning.

### 4.4 Association between symptom distress and quality of life in patients with CKD Stage 5 undergoing hemodialysis

Spearman rank correlation analysis demonstrated that the total DSI score was significantly negatively correlated with the majority of domains of the KDQOL-SF™ 1.3 (*P* < 0.001), indicating that greater symptom distress was associated with poorer quality of life. These findings are consistent with the conclusions of the systematic review and meta-analysis conducted by Lu et al. (38) and van Oevelen et al. (39).

Among all domains, the Symptom/Problem List demonstrated the strongest correlation with the total DSI score (*r* = -0.890), reaching a very strong negative correlation. This finding provides mutual validation of the two instruments in assessing symptom burden. As a symptom-specific measure, the DSI showed high concordance with the established symptom-related domain of the KDQOL-SF™ 1.3, thereby supporting the reliability and consistency of both instruments in the study population.

Within the Kidney Disease-Targeted Dimensions, Effects of Kidney Disease (*r* = -0.693), Cognitive Function (*r* = -0.578), Sexual Function (*r* = -0.548), and Sleep (*r* = -0.492) demonstrated moderate to strong negative correlations with the total DSI score. These findings indicate that symptom distress extends beyond the physical domain and permeates cognitive functioning, sexual functioning, and sleep, thereby contributing to multidimensional impairments in quality of life.

Among the SF-36 Generic Core Dimensions, Energy/Fatigue (*r* = -0.594), Pain (*r* = -0.578), Physical Functioning (*r* = -0.559), and Social Functioning (*r* = -0.522) showed relatively strong correlations with the total DSI score. Notably, the strong association between the Energy/Fatigue domain and overall symptom distress suggests that fatigue may be a key determinant of patients’ quality of life. Similarly, the strong correlation observed in the Pain domain further underscores the critical importance of effective pain management in improving quality of life among patients undergoing hemodialysis.

It is noteworthy that Dialysis Staff Encouragement (*r* = -0.177) and Patient Satisfaction (*r* = -0.131), although significantly correlated with the total DSI score, showed relatively low correlation coefficients. This suggests that while healthcare provider support and patient satisfaction may indirectly alleviate symptom distress—by improving emotional experiences and treatment adherence—their direct impact on symptom burden appears limited. In other words, the primary value of healthcare support lies in helping patients better cope with their illness and maintain emotional well-being, whereas the alleviation of symptoms themselves still depends largely on targeted treatment of underlying causes.

After adjustment for potential confounding factors using multiple linear regression analysis, the total DSI score remained an independent predictor of all KDQOL-SF™ 1.3 domains except Patient Satisfaction (*P* < 0.05). This finding further reinforces the negative association between symptom distress and quality of life, indicating that symptom assessment and management should be regarded as central therapeutic targets for improving quality of life in clinical practice.

In addition, this study examined the associations between dialysis adequacy indicators (Kt/V and URR) and various domains of quality of life. Multiple linear regression analysis showed that, after adjusting for potential confounders, Kt/V was independently and positively associated with Quality of Social Interaction (B = 8.215, *P* = 0.031) and Pain (B = 8.527, *P* = 0.041), suggesting that higher Kt/V levels may contribute to better social functioning and reduced pain. However, neither Kt/V nor URR was significantly associated with the other quality-of-life domains, nor were they significantly correlated with the total DSI score. These findings indicate that the relationship between dialysis adequacy parameters and patient-reported outcomes is complex. Further research is warranted to clarify the underlying mechanisms of these associations.

### 4.5 Limitations

This study has several noteworthy limitations.

The sample size of this study was 359 patients, representing a moderate scale compared with other hemodialysis studies conducted both domestically and internationally. While this sample size was adequate to meet the primary research objective—namely, multiple linear regression analysis—it remains relatively limited for more in-depth analyses of certain subgroups, such as patients with lower educational levels or specific primary disease types.

Second, as a cross-sectional study, this research can only identify associations between symptom distress and quality of life, but cannot infer causal relationships or determine the directionality of these associations. Moreover, it is unable to assess the dynamic changes in symptom distress and quality of life over the course of the disease or over time (40, 41).

Third, this study excluded patients who were unable to complete the survey. Although this approach helped ensure the reliability of the questionnaire data, it may have led to an underestimation of symptom distress in this subgroup—particularly among those with severe cognitive impairment or communication difficulties—thereby limiting the generalizability of the findings to the broader hemodialysis population. Given that previous studies have demonstrated a high prevalence of cognitive impairment among patients undergoing hemodialysis, future research should consider developing simplified assessment tools tailored for cognitively impaired patients or employing alternative approaches such as pictorial questionnaires or interviewer-administered assessments. These strategies may help more comprehensively capture the health status and subjective experiences of this vulnerable population (42–44).

Finally, this study primarily relied on patient-reported instruments (the DSI and KDQOL-SF™ 1.3), and the findings may therefore be influenced by subjective factors inherent to self-report measures (45). Future multicenter studies with larger sample sizes and longitudinal follow-up are warranted to validate the present findings and to further explore the dynamic changes in symptom distress and quality of life over time.

## 5 Conclusions

This study described the current status of symptom distress and quality of life among Chinese patients with CKD Stage 5 undergoing hemodialysis and explored the association between these two variables.

Patients experienced a substantial symptom burden, reporting an average of 13 symptoms, with sexual dysfunction, sleep disturbances, and emotional and cognitive symptoms being the most prevalent. Independent predictors of symptom distress included older age, longer dialysis vintage, lower educational level, and diabetic nephropathy as the primary disease. Regarding quality of life, patients had relatively low scores in domains such as Work Status, Burden of Kidney Disease, Role Limitations - Physical, Change in Health, and General Health Perceptions. In contrast, higher scores were observed in Dialysis Staff Encouragement and Role Limitations - Emotional, suggesting that although physical functioning is markedly limited, effective healthcare support may help preserve patients’ psychological well-being.

Both Spearman rank correlation analysis and multiple linear regression analysis confirmed that the severity of symptom distress was significantly negatively associated with all domains of quality of life (except Patient Satisfaction), with the strongest correlation observed for the Symptom/Problem List (*r* = -0.890). These findings indicate that symptom distress is a key determinant of quality of life in this population, and that inadequate symptom control is closely linked to poorer quality of life outcomes.

This study provides multicenter data on symptom distress and quality of life among Chinese patients with CKD Stage 5 undergoing hemodialysis. The findings highlight the need for routine symptom screening and comprehensive symptom management in clinical practice, with particular attention to patients who are older, have longer dialysis vintage, lower educational levels, or diabetic nephropathy. Future research should adopt longitudinal designs to further validate the association between symptom distress and quality of life and to explore their dynamic trajectories over time.

## Data Availability

The raw data supporting the conclusions of this article will be made available by the authors, without undue reservation.
