# Supplementary material for: Correlation between symptom distress and quality of life in patients with Chronic Kidney Disease Stage 5 undergoing hemodialysis in China: a multi-center cross-sectional study
Source: Front Psychiatry. 2026 Mar 27;17:1733502. doi: 10.3389/fpsyt.2026.1733502 (PMC13066137; doi:10.3389/fpsyt.2026.1733502)
Supplement: Supplementary file 1 [file Table1.docx]

**Table S1. Multiple Linear Regression Analysis of the Total DSI Score and the Domain Scores of the KDQOL-SF™ 1.3.**

| Variable | Symptom/Problem List | | | Effects of Kidney Disease | | | Burden of Kidney Disease | | | Work Status | | | Cognitive Function | | | Quality of Social Interaction | | | Sexual Function | | |
| --- | --- | --- | --- | --- | --- | --- | --- | --- | --- | --- | --- | --- | --- | --- | --- | --- | --- | --- | --- | --- | --- |
|  | B | P | VIF | B | P | VIF | B | P | VIF | B | P | VIF | B | P | VIF | B | P | VIF | B | P | VIF |
| Total DSI score | -1.197 | <0.001 | 1.340 | -1.328 | <0.001 | 1.340 | -1.080 | <0.001 | 1.340 | -0.534 | 0.004 | 1.34 | -1.093 | <0.001 | 1.340 | -0.878 | <0.001 | 1.340 | -1.834 | 0.002 | 3.395 |
| **Sex** |  | | |  | | |  | | |  | | |  | | |  | | |  | | |
| Female | 0.776 | 0.326 | 1.380 | 3.954 | 0.029 | 1.380 | 5.164 | 0.056 | 1.380 | 7.271 | 0.071 | 1.38 | 2.385 | 0.192 | 1.380 | 1.545 | 0.402 | 1.380 | 14.157 | 0.227 | 2.889 |
| Male | 0 |  |  | 0 |  |  | 0 |  |  | 0 |  |  | 0 |  |  | 0 |  |  | 0 |  |  |
| Age | -0.004 | 0.905 | 1.750 | 0.101 | 0.221 | 1.750 | 0.047 | 0.701 | 1.750 | 0.07 | 0.703 | 1.75 | 0.191 | 0.023 | 1.750 | 0.211 | 0.013 | 1.750 | -0.397 | 0.451 | 2.522 |
| **Education level** |  | | |  | | |  | | |  | | |  | | |  | | |  | | |
| Junior high school/Senior high school/Technical secondary school/Vocational high school | -0.535 | 0.742 | 5.547 | 1.473 | 0.691 | 5.547 | -1.794 | 0.746 | 5.547 | 10.13 | 0.220 | 5.547 | 2.739 | 0.465 | 5.547 | 2.432 | 0.521 | 5.547 | -3.865 | 0.916 | 31.153 |
| Junior college/Bachelor’s degree or above | -1.224 | 0.478 | 5.862 | -0.152 | 0.969 | 5.862 | -3.550 | 0.546 | 5.862 | 15.576 | 0.076 | 5.862 | 3.527 | 0.376 | 5.862 | 3.708 | 0.357 | 5.862 | 19.633 | 0.592 | 29.923 |
| Primary school or below | 0 |  |  | 0 |  |  | 0 |  |  | 0 |  |  | 0 |  |  | 0 |  |  | 0 |  |  |
| **Marital status** |  | | |  | | |  | | |  | | |  | | |  | | |  | | |
| Unmarried | 1.522 | 0.149 | 1.195 | -1.697 | 0.480 | 1.195 | -2.511 | 0.485 | 1.195 | -7.131 | 0.184 | 1.195 | 2.357 | 0.333 | 1.195 | -3.706 | 0.132 | 1.195 | 4.126 | 0.828 | 1.630 |
| Married | 0 |  |  | 0 |  |  | 0 |  |  | 0 |  |  | 0 |  |  | 0 |  |  | 0 |  |  |
| **Employment status** |  | | |  | | |  | | |  | | |  | | |  | | |  | | |
| Unemployed | 0.026 | 0.974 | 1.424 | -1.680 | 0.360 | 1.424 | 1.557 | 0.570 | 1.424 | -7.672 | 0.061 | 1.424 | 0.331 | 0.859 | 1.424 | 0.548 | 0.770 | 1.424 | 10.639 | 0.330 | 2.532 |
| Employed | 0 |  |  | 0 |  |  | 0 |  |  | 0 |  |  | 0 |  |  | 0 |  |  | 0 |  |  |
| BMI | -0.068 | 0.486 | 1.425 | 0.042 | 0.850 | 1.425 | -0.48 | 0.149 | 1.425 | 0.08 | 0.871 | 1.425 | -0.306 | 0.175 | 1.425 | -0.440 | 0.053 | 1.425 | -0.999 | 0.399 | 2.972 |
| Dialysis vintage | -0.013 | 0.019 | 1.520 | -0.003 | 0.796 | 1.520 | -0.017 | 0.375 | 1.52 | -0.034 | 0.225 | 1.52 | -0.004 | 0.761 | 1.520 | -0.001 | 0.947 | 1.520 | 0.115 | 0.124 | 2.130 |
| **Dialysis frequency** |  | | |  | | |  | | |  | | |  | | |  | | |  | | |
| Non–3-times-per-week | 0.303 | 0.801 | 1.141 | 2.196 | 0.423 | 1.141 | 1.464 | 0.721 | 1.141 | 6.913 | 0.259 | 1.141 | 0.252 | 0.928 | 1.141 | 0.854 | 0.761 | 1.141 | 2.511 | 0.833 | 1.710 |
| 3 times per week | 0 |  |  | 0 |  |  | 0 |  |  | 0 |  |  | 0 |  |  | 0 |  |  | 0 |  |  |
| Kt/V | 1.080 | 0.506 | 3.975 | -3.978 | 0.283 | 3.975 | -4.986 | 0.368 | 3.975 | 5.083 | 0.538 | 3.975 | 1.721 | 0.647 | 3.975 | 8.215 | 0.031 | 3.975 | 12.654 | 0.691 | 16.964 |
| URR | 0.025 | 0.797 | 5.118 | 0.288 | 0.196 | 5.118 | 0.439 | 0.188 | 5.118 | -0.561 | 0.259 | 5.118 | -0.072 | 0.751 | 5.118 | -0.377 | 0.099 | 5.118 | -2.022 | 0.267 | 20.631 |
| **Dialysis modality** |  | | |  | | |  | | |  | | |  | | |  | | |  | | |
| Conventional | 1.521 | 0.096 | 1.853 | 0.069 | 0.973 | 1.853 | 4.994 | 0.109 | 1.853 | 15.204 | 0.001 | 1.853 | 3.456 | 0.102 | 1.853 | 9.752 | <0.001 | 1.853 | 9.899 | 0.544 | 6.275 |
| High-flux | 0 |  |  | 0 |  |  | 0 |  |  | 0 |  |  | 0 |  |  | 0 |  |  | 0 |  |  |
| **Primary disease** |  | | |  | | |  | | |  | | |  | | |  | | |  | | |
| Diabetic nephropathy | -0.082 | 0.938 | 1.975 | -0.561 | 0.814 | 1.975 | 0.247 | 0.945 | 1.975 | -7.291 | 0.171 | 1.975 | 7.589 | 0.002 | 1.975 | 2.813 | 0.250 | 1.975 | 10.144 | 0.474 | 3.690 |
| Hypertensive nephropathy | 0.580 | 0.565 | 1.636 | 0.812 | 0.724 | 1.636 | 10.558 | 0.002 | 1.636 | -0.475 | 0.926 | 1.636 | 4.623 | 0.048 | 1.636 | 1.304 | 0.579 | 1.636 | 5.036 | 0.647 | 1.946 |
| Other causes | -1.061 | 0.314 | 1.548 | 2.827 | 0.240 | 1.548 | 4.948 | 0.169 | 1.548 | 2.205 | 0.681 | 1.548 | 7.234 | 0.003 | 1.548 | 4.469 | 0.070 | 1.548 | 25.303 | 0.096 | 2.161 |
| Chronic glomerulonephritis | 0 |  |  | 0 |  |  | 0 |  |  | 0 |  |  | 0 |  |  | 0 |  |  | 0 |  |  |
| **Comorbidities** |  | | |  | | |  | | |  | | |  | | |  | | |  | | |
| Hyperlipidemia | 1.278 | 0.275 | 1.519 | -2.605 | 0.329 | 1.519 | -2.585 | 0.517 | 1.519 | -1.448 | 0.808 | 1.519 | -1.278 | 0.636 | 1.519 | -6.315 | 0.021 | 1.519 | 12.296 | 0.617 | 3.558 |
| Ischemic heart disease | -0.428 | 0.688 | 1.362 | 2.503 | 0.304 | 1.362 | 3.83 | 0.293 | 1.362 | 3.376 | 0.534 | 1.362 | 4.200 | 0.089 | 1.362 | -0.569 | 0.819 | 1.362 | 9.477 | 0.544 | 2.368 |
| Diabetes mellitus | 0.589 | 0.557 | 1.389 | -0.601 | 0.793 | 1.389 | 0.646 | 0.850 | 1.389 | -2.29 | 0.654 | 1.389 | -1.805 | 0.437 | 1.389 | 3.411 | 0.146 | 1.389 | 15.119 | 0.467 | 3.103 |
| Other comorbidities | -0.292 | 0.837 | 1.254 | -0.784 | 0.808 | 1.254 | -1.021 | 0.833 | 1.254 | -6.658 | 0.356 | 1.254 | 0.726 | 0.825 | 1.254 | 3.146 | 0.341 | 1.254 | 17.497 | 0.493 | 1.979 |
| Hypertension | 0 |  |  | 0 |  |  | 0 |  |  | 0 |  |  | 0 |  |  | 0 |  |  | 0 |  |  |
| **Presence of comorbidities** |  | | |  | | |  | | |  | | |  | | |  | | |  | | |
| No | -0.470 | 0.631 | 1.740 | -1.058 | 0.635 | 1.740 | -6.476 | 0.052 | 1.740 | -4.189 | 0.399 | 1.740 | -1.722 | 0.446 | 1.740 | -3.120 | 0.171 | 1.740 | 7.754 | 0.460 | 2.589 |
| Yes | 0 |  |  | 0 |  |  | 0 |  |  | 0 |  |  | 0 |  |  | 0 |  |  | 0 |  |  |
| **Complications** |  | | |  | | |  | | |  | | |  | | |  | | |  | | |
| CKD-MBD | 1.115 | 0.242 | 1.502 | 4.526 | 0.038 | 1.502 | -1.785 | 0.582 | 1.502 | -8.378 | 0.084 | 1.502 | -0.783 | 0.721 | 1.502 | -1.421 | 0.522 | 1.502 | -7.345 | 0.583 | 2.708 |
| Renal hypertension | 0.896 | 0.279 | 1.492 | -1.019 | 0.589 | 1.492 | -1.533 | 0.586 | 1.492 | -8.238 | 0.050 | 1.492 | -0.814 | 0.670 | 1.492 | -1.454 | 0.450 | 1.492 | -32.501 | 0.047 | 5.390 |
| Malnutrition | 0.368 | 0.731 | 1.123 | 0.387 | 0.874 | 1.123 | 0.937 | 0.797 | 1.123 | -4.946 | 0.363 | 1.123 | 3.782 | 0.126 | 1.123 | 0.765 | 0.759 | 1.123 | 14.108 | 0.256 | 1.994 |
| Intradialytic hypotension | 2.041 | 0.089 | 1.411 | -0.837 | 0.759 | 1.411 | -8.277 | 0.043 | 1.411 | -9.304 | 0.127 | 1.411 | -3.145 | 0.256 | 1.411 | -1.733 | 0.535 | 1.411 | -30.833 | 0.037 | 3.079 |
| Hyperuricemia | -0.884 | 0.476 | 1.482 | 1.658 | 0.558 | 1.482 | 0.752 | 0.859 | 1.482 | 0.305 | 0.961 | 1.482 | 3.102 | 0.279 | 1.482 | -1.970 | 0.495 | 1.482 | -16.435 | 0.413 | 2.890 |
| Other complications | -3.231 | 0.074 | 1.244 | 4.385 | 0.287 | 1.244 | 14.66 | 0.018 | 1.244 | 5.794 | 0.528 | 1.244 | 7.628 | 0.068 | 1.244 | 3.525 | 0.402 | 1.244 | 52.660 | 0.210 | 2.679 |
| Renal anemia | 0 |  |  | 0 |  |  | 0 |  |  | 0 |  |  | 0 |  |  | 0 |  |  | 0 |  |  |
| **Polypharmacy status** |  | | |  | | |  | | |  | | |  | | |  | | |  | | |
| Polypharmacy | 0.813 | 0.392 | 2.003 | 1.276 | 0.556 | 2.003 | -3.659 | 0.259 | 2.003 | -9.834 | 0.042 | 2.003 | -0.252 | 0.908 | 2.003 | -3.426 | 0.122 | 2.003 | -7.357 | 0.544 | 2.822 |
| Excessive polypharmacy | 1.695 | 0.162 | 2.537 | 1.466 | 0.595 | 2.537 | -0.356 | 0.931 | 2.537 | -0.407 | 0.947 | 2.537 | 2.366 | 0.398 | 2.537 | 0.218 | 0.938 | 2.537 | 11.889 | 0.547 | 4.698 |
| Non-polypharmacy | 0 |  |  | 0 |  |  | 0 |  |  | 0 |  |  | 0 |  |  | 0 |  |  | 0 |  |  |
| R^2^ | 0.824 | | | 0.526 | | | 0.315 | | | 0.177 | | | 0.431 | | | 0.386 | | | 0.688 | | |
| Durbin–Watson | 1.831 | | | 1.886 | | | 1.902 | | | 1.867 | | | 2.122 | | | 2.009 | | | 2.313 | | |
| Variable | Sleep | | | Social Support | | | Dialysis Staff Encouragement | | | Patient Satisfaction | | | Physical Functioning | | | Role Limitations - Physical | | | Role Limitations - Emotional | | |
|  | B | P | VIF | B | P | VIF | B | P | VIF | B | P | VIF | B | P | VIF | B | P | VIF | B | P | VIF |
| Total DSI score | -0.799 | <0.001 | 1.340 | -0.433 | <0.001 | 1.340 | -0.225 | 0.009 | 1.340 | -0.153 | 0.137 | 1.344 | -1.008 | <0.001 | 1.340 | -1.601 | <0.001 | 1.340 | -1.911 | <0.001 | 1.340 |
| **Sex** |  | | |  | | |  | | |  | | |  | | |  | | |  | | |
| Female | 1.686 | 0.437 | 1.380 | -0.079 | 0.977 | 1.380 | -0.299 | 0.873 | 1.380 | 0.416 | 0.853 | 1.389 | -3.589 | 0.140 | 1.380 | 8.155 | 0.091 | 1.380 | 7.521 | 0.104 | 1.380 |
| Male | 0 |  |  | 0 |  |  | 0 |  |  | 0 |  |  | 0 |  |  | 0 |  |  | 0 |  |  |
| Age | -0.284 | 0.004 | 1.750 | 0.092 | 0.470 | 1.750 | 0.088 | 0.302 | 1.750 | -0.149 | 0.145 | 1.754 | -0.543 | <0.001 | 1.750 | -0.732 | <0.001 | 1.750 | -0.535 | 0.012 | 1.750 |
| **Education level** |  | | |  | | |  | | |  | | |  | | |  | | |  | | |
| Junior high school/Senior high school/Technical secondary school/Vocational high school | 3.802 | 0.393 | 5.547 | 1.905 | 0.739 | 5.547 | -3.157 | 0.411 | 5.547 | -2.467 | 0.590 | 5.522 | 3.671 | 0.461 | 5.547 | 7.962 | 0.421 | 5.547 | -2.462 | 0.795 | 5.547 |
| Junior college/Bachelor’s degree or above | 5.833 | 0.218 | 5.862 | 6.981 | 0.251 | 5.862 | -5.754 | 0.159 | 5.862 | -1.731 | 0.722 | 5.830 | 0.099 | 0.985 | 5.862 | -5.559 | 0.597 | 5.862 | -6.213 | 0.538 | 5.862 |
| Primary school or below | 0 |  |  | 0 |  |  | 0 |  |  | 0 |  |  | 0 |  |  | 0 |  |  | 0 |  |  |
| **Marital status** |  | | |  | | |  | | |  | | |  | | |  | | |  | | |
| Unmarried | 0.102 | 0.972 | 1.195 | -8.085 | 0.030 | 1.195 | 0.307 | 0.902 | 1.195 | -2.708 | 0.363 | 1.195 | -7.383 | 0.023 | 1.195 | -18.024 | 0.005 | 1.195 | -6.725 | 0.276 | 1.195 |
| Married | 0 |  |  | 0 |  |  | 0 |  |  | 0 |  |  | 0 |  |  | 0 |  |  | 0 |  |  |
| **Employment status** |  | | |  | | |  | | |  | | |  | | |  | | |  | | |
| Unemployed | 4.169 | 0.059 | 1.424 | 1.335 | 0.638 | 1.424 | -1.610 | 0.397 | 1.424 | -1.074 | 0.637 | 1.430 | -3.961 | 0.109 | 1.424 | -1.886 | 0.700 | 1.424 | 7.392 | 0.117 | 1.424 |
| Employed | 0 |  |  | 0 |  |  | 0 |  |  | 0 |  |  | 0 |  |  | 0 |  |  | 0 |  |  |
| BMI | 0.043 | 0.872 | 1.425 | -0.245 | 0.476 | 1.425 | -0.274 | 0.235 | 1.425 | 0.085 | 0.759 | 1.436 | -0.920 | 0.002 | 1.425 | -0.952 | 0.109 | 1.425 | -1.101 | 0.054 | 1.425 |
| Dialysis vintage | -0.005 | 0.754 | 1.520 | -0.004 | 0.834 | 1.520 | -0.002 | 0.907 | 1.520 | -0.023 | 0.142 | 1.515 | -0.035 | 0.041 | 1.520 | 0.001 | 0.987 | 1.520 | -0.016 | 0.630 | 1.520 |
| **Dialysis frequency** |  | | |  | | |  | | |  | | |  | | |  | | |  | | |
| Non–3-times-per-week | -1.879 | 0.569 | 1.141 | -7.569 | 0.075 | 1.141 | 3.236 | 0.256 | 1.141 | 5.334 | 0.116 | 1.141 | -0.245 | 0.947 | 1.141 | -0.722 | 0.921 | 1.141 | -10.913 | 0.121 | 1.141 |
| 3 times per week | 0 |  |  | 0 |  |  | 0 |  |  | 0 |  |  | 0 |  |  | 0 |  |  | 0 |  |  |
| Kt/V | -2.663 | 0.550 | 3.975 | -1.392 | 0.808 | 3.975 | 1.711 | 0.656 | 3.975 | -0.926 | 0.840 | 3.975 | 2.972 | 0.551 | 3.975 | -4.341 | 0.661 | 3.975 | 0.531 | 0.955 | 3.975 |
| URR | 0.355 | 0.185 | 5.118 | 0.503 | 0.144 | 5.118 | -0.177 | 0.444 | 5.118 | 0.243 | 0.378 | 5.125 | -0.415 | 0.167 | 5.118 | -0.178 | 0.765 | 5.118 | -0.100 | 0.862 | 5.118 |
| **Dialysis modality** |  | | |  | | |  | | |  | | |  | | |  | | |  | | |
| Conventional | 4.362 | 0.082 | 1.853 | 10.726 | <0.001 | 1.853 | 8.777 | <0.001 | 1.853 | 9.830 | <0.001 | 1.847 | -1.866 | 0.505 | 1.853 | 6.633 | 0.233 | 1.853 | 11.584 | 0.030 | 1.853 |
| High-flux | 0 |  |  | 0 |  |  | 0 |  |  | 0 |  |  | 0 |  |  | 0 |  |  | 0 |  |  |
| **Primary disease** |  | | |  | | |  | | |  | | |  | | |  | | |  | | |
| Diabetic nephropathy | 0.290 | 0.919 | 1.975 | -0.431 | 0.907 | 1.975 | 0.631 | 0.799 | 1.975 | -0.229 | 0.938 | 1.968 | -12.356 | <0.001 | 1.975 | -8.589 | 0.178 | 1.975 | -2.450 | 0.689 | 1.975 |
| Hypertensive nephropathy | 0.095 | 0.972 | 1.636 | 0.758 | 0.831 | 1.636 | -3.997 | 0.094 | 1.636 | -2.948 | 0.300 | 1.635 | -1.423 | 0.646 | 1.636 | -10.650 | 0.084 | 1.636 | -1.227 | 0.835 | 1.636 |
| Other causes | -0.898 | 0.756 | 1.548 | -6.342 | 0.088 | 1.548 | -0.972 | 0.697 | 1.548 | -2.417 | 0.416 | 1.547 | 0.419 | 0.897 | 1.548 | -13.267 | 0.039 | 1.548 | 4.696 | 0.446 | 1.548 |
| Chronic glomerulonephritis | 0 |  |  | 0 |  |  | 0 |  |  | 0 |  |  | 0 |  |  | 0 |  |  | 0 |  |  |
| **Comorbidities** |  | | |  | | |  | | |  | | |  | | |  | | |  | | |
| Hyperlipidemia | -2.141 | 0.504 | 1.519 | -9.783 | 0.018 | 1.519 | -3.263 | 0.238 | 1.519 | -6.395 | 0.054 | 1.502 | -4.981 | 0.166 | 1.519 | -8.923 | 0.211 | 1.519 | -5.066 | 0.459 | 1.519 |
| Ischemic heart disease | -1.996 | 0.495 | 1.362 | -4.233 | 0.260 | 1.362 | 2.039 | 0.419 | 1.362 | -2.258 | 0.454 | 1.365 | -1.917 | 0.558 | 1.362 | 8.267 | 0.203 | 1.362 | -2.712 | 0.663 | 1.362 |
| Diabetes mellitus | 2.465 | 0.371 | 1.389 | 2.434 | 0.491 | 1.389 | -1.766 | 0.457 | 1.389 | -1.802 | 0.526 | 1.380 | -6.140 | 0.047 | 1.389 | 0.908 | 0.882 | 1.389 | 4.689 | 0.425 | 1.389 |
| Other comorbidities | 3.376 | 0.385 | 1.254 | -1.859 | 0.710 | 1.254 | 1.857 | 0.580 | 1.254 | 1.702 | 0.670 | 1.254 | -2.324 | 0.593 | 1.254 | -6.499 | 0.452 | 1.254 | 5.982 | 0.471 | 1.254 |
| Hypertension | 0 |  |  | 0 |  |  | 0 |  |  | 0 |  |  | 0 |  |  | 0 |  |  | 0 |  |  |
| **Presence of comorbidities** |  | | |  | | |  | | |  | | |  | | |  | | |  | | |
| No | -1.092 | 0.683 | 1.740 | 2.113 | 0.539 | 1.740 | -4.196 | 0.070 | 1.740 | -3.884 | 0.159 | 1.738 | -0.148 | 0.961 | 1.740 | -1.866 | 0.754 | 1.740 | -14.008 | 0.015 | 1.740 |
| Yes | 0 |  |  | 0 |  |  | 0 |  |  | 0 |  |  | 0 |  |  | 0 |  |  | 0 |  |  |
| **Complications** |  | | |  | | |  | | |  | | |  | | |  | | |  | | |
| CKD-MBD | 0.289 | 0.912 | 1.502 | 2.071 | 0.537 | 1.502 | -1.150 | 0.609 | 1.502 | 0.283 | 0.916 | 1.502 | 8.430 | 0.004 | 1.502 | 0.319 | 0.956 | 1.502 | 0.799 | 0.886 | 1.502 |
| Renal hypertension | 5.082 | 0.025 | 1.492 | 7.781 | 0.008 | 1.492 | 3.987 | 0.042 | 1.492 | 1.273 | 0.585 | 1.489 | 1.480 | 0.559 | 1.492 | 8.027 | 0.111 | 1.492 | 0.274 | 0.955 | 1.492 |
| Malnutrition | -3.335 | 0.255 | 1.123 | 8.059 | 0.033 | 1.123 | 0.396 | 0.875 | 1.123 | -2.054 | 0.496 | 1.123 | 3.117 | 0.342 | 1.123 | 7.936 | 0.223 | 1.123 | 2.002 | 0.749 | 1.123 |
| Intradialytic hypotension | 6.417 | 0.051 | 1.411 | -2.429 | 0.565 | 1.411 | -2.322 | 0.413 | 1.411 | -1.239 | 0.714 | 1.411 | 0.131 | 0.972 | 1.411 | -4.465 | 0.541 | 1.411 | -4.290 | 0.540 | 1.411 |
| Hyperuricemia | 4.440 | 0.192 | 1.482 | 5.529 | 0.206 | 1.482 | -3.424 | 0.243 | 1.482 | -2.437 | 0.487 | 1.461 | -4.051 | 0.287 | 1.482 | 14.124 | 0.062 | 1.482 | 18.323 | 0.012 | 1.482 |
| Other complications | -1.605 | 0.746 | 1.244 | 1.382 | 0.828 | 1.244 | 3.244 | 0.447 | 1.244 | 9.900 | 0.052 | 1.245 | 16.600 | 0.003 | 1.244 | 14.694 | 0.182 | 1.244 | 19.371 | 0.067 | 1.244 |
| Renal anemia | 0 |  |  | 0 |  |  | 0 |  |  | 0 |  |  | 0 |  |  | 0 |  |  | 0 |  |  |
| **Polypharmacy status** |  | | |  | | |  | | |  | | |  | | |  | | |  | | |
| Polypharmacy | -5.004 | 0.055 | 2.003 | -0.701 | 0.834 | 2.003 | -1.635 | 0.466 | 2.003 | -2.738 | 0.306 | 1.997 | -1.536 | 0.598 | 2.003 | 0.526 | 0.927 | 2.003 | 1.008 | 0.856 | 2.003 |
| Excessive polypharmacy | -3.582 | 0.280 | 2.537 | -4.274 | 0.316 | 2.537 | -2.010 | 0.483 | 2.537 | -1.522 | 0.656 | 2.517 | -4.596 | 0.216 | 2.537 | -3.835 | 0.603 | 2.537 | 6.254 | 0.377 | 2.537 |
| Non-polypharmacy | 0 |  |  | 0 |  |  | 0 |  |  | 0 |  |  | 0 |  |  | 0 |  |  | 0 |  |  |
| R2 | 0.309 | | | 0.219 | | | 0.165 | | | 0.139 | | | 0.452 | | | 0.326 | | | 0.327 | | |
| Durbin–Watson | 1.834 | | | 2.051 | | | 1.899 | | | 1.838 | | | 1.998 | | | 1.881 | | | 2.003 | | |
| Variable | Social Functioning | | | Emotional Well-Being | | | Pain | | | Energy/Fatigue | | | General Health Perceptions | | | Overall Health Rating | | | Change in Health | | |
|  | B | P | VIF | B | P | VIF | B | P | VIF | B | P | VIF | B | P | VIF | B | P | VIF | B | P | VIF |
| Total DSI score | -1.272 | <0.001 | 1.340 | -0.853 | <0.001 | 1.340 | -1.173 | <0.001 | 1.340 | -1.044 | <0.001 | 1.340 | -0.880 | <0.001 | 1.340 | -0.722 | <0.001 | 1.340 | -0.730 | <0.001 | 1.340 |
| **Sex** |  | | |  | | |  | | |  | | |  | | |  | | |  | | |
| Female | 4.995 | 0.051 | 1.380 | 1.329 | 0.526 | 1.380 | -5.724 | 0.005 | 1.380 | 3.949 | 0.051 | 1.380 | 0.331 | 0.875 | 1.380 | 3.296 | 0.140 | 1.380 | -1.285 | 0.691 | 1.380 |
| Male | 0 |  |  | 0 |  |  | 0 |  |  | 0 |  |  | 0 |  |  | 0 |  |  | 0 |  |  |
| Age | -0.110 | 0.348 | 1.750 | 0.097 | 0.311 | 1.750 | -0.126 | 0.174 | 1.750 | 0.031 | 0.739 | 1.750 | 0.061 | 0.526 | 1.750 | 0.183 | 0.073 | 1.750 | 0.037 | 0.801 | 1.750 |
| **Education level** |  | | |  | | |  | | |  | | |  | | |  | | |  | | |
| Junior high school/Senior high school/Technical secondary school/Vocational high school | -6.610 | 0.208 | 5.547 | -9.433 | 0.029 | 5.547 | -6.119 | 0.142 | 5.547 | -2.719 | 0.511 | 5.547 | -8.071 | 0.062 | 5.547 | 2.184 | 0.634 | 5.547 | -9.394 | 0.157 | 5.547 |
| Junior college/Bachelor’s degree or above | -7.492 | 0.180 | 5.862 | -8.980 | 0.050 | 5.862 | -7.582 | 0.087 | 5.862 | -1.595 | 0.717 | 5.862 | -9.297 | 0.043 | 5.862 | -1.252 | 0.797 | 5.862 | -15.036 | 0.033 | 5.862 |
| Primary school or below | 0 |  |  | 0 |  |  | 0 |  |  | 0 |  |  | 0 |  |  | 0 |  |  | 0 |  |  |
| **Marital status** |  | | |  | | |  | | |  | | |  | | |  | | |  | | |
| Unmarried | -3.958 | 0.246 | 1.195 | 0.131 | 0.963 | 1.195 | 3.810 | 0.159 | 1.195 | -4.739 | 0.079 | 1.195 | -3.024 | 0.281 | 1.195 | 2.184 | 0.463 | 1.195 | -5.385 | 0.212 | 1.195 |
| Married | 0 |  |  | 0 |  |  | 0 |  |  | 0 |  |  | 0 |  |  | 0 |  |  | 0 |  |  |
| **Employment status** |  | | |  | | |  | | |  | | |  | | |  | | |  | | |
| Unemployed | -0.536 | 0.837 | 1.424 | 0.014 | 0.995 | 1.424 | 0.685 | 0.740 | 1.424 | 1.015 | 0.621 | 1.424 | 2.843 | 0.184 | 1.424 | -0.653 | 0.774 | 1.424 | 0.044 | 0.989 | 1.424 |
| Employed | 0 |  |  | 0 |  |  | 0 |  |  | 0 |  |  | 0 |  |  | 0 |  |  | 0 |  |  |
| BMI | -0.568 | 0.072 | 1.425 | -0.149 | 0.565 | 1.425 | -0.958 | <0.001 | 1.425 | -0.170 | 0.494 | 1.425 | -0.125 | 0.630 | 1.425 | 0.160 | 0.561 | 1.425 | -0.572 | 0.151 | 1.425 |
| Dialysis vintage | 0.005 | 0.783 | 1.520 | -0.001 | 0.939 | 1.520 | -0.042 | 0.004 | 1.520 | 0.002 | 0.895 | 1.520 | -0.019 | 0.206 | 1.520 | -0.003 | 0.865 | 1.520 | -0.109 | <0.001 | 1.520 |
| **Dialysis frequency** |  | | |  | | |  | | |  | | |  | | |  | | |  | | |
| Non–3-times-per-week | -0.831 | 0.831 | 1.141 | -0.502 | 0.875 | 1.141 | 1.512 | 0.624 | 1.141 | -0.472 | 0.878 | 1.141 | 1.688 | 0.598 | 1.141 | 2.079 | 0.540 | 1.141 | 12.819 | 0.009 | 1.141 |
| 3 times per week | 0 |  |  | 0 |  |  | 0 |  |  | 0 |  |  | 0 |  |  | 0 |  |  | 0 |  |  |
| Kt/V | 6.807 | 0.195 | 3.975 | 2.978 | 0.489 | 3.975 | 8.527 | 0.041 | 3.975 | -2.290 | 0.580 | 3.975 | -2.334 | 0.589 | 3.975 | -4.523 | 0.324 | 3.975 | -0.154 | 0.982 | 3.975 |
| URR | -0.333 | 0.293 | 5.118 | -0.036 | 0.890 | 5.118 | -0.399 | 0.112 | 5.118 | 0.170 | 0.496 | 5.118 | 0.183 | 0.480 | 5.118 | 0.543 | 0.050 | 5.118 | 0.029 | 0.941 | 5.118 |
| **Dialysis modality** |  | | |  | | |  | | |  | | |  | | |  | | |  | | |
| Conventional | 4.704 | 0.111 | 1.853 | 5.511 | 0.023 | 1.853 | 1.803 | 0.441 | 1.853 | 6.196 | 0.008 | 1.853 | 0.281 | 0.908 | 1.853 | 7.083 | 0.006 | 1.853 | -11.877 | 0.002 | 1.853 |
| High-flux | 0 |  |  | 0 |  |  | 0 |  |  | 0 |  |  | 0 |  |  | 0 |  |  | 0 |  |  |
| **Primary disease** |  | | |  | | |  | | |  | | |  | | |  | | |  | | |
| Diabetic nephropathy | -2.913 | 0.390 | 1.975 | -1.162 | 0.675 | 1.975 | -0.198 | 0.941 | 1.975 | -2.243 | 0.401 | 1.975 | 0.385 | 0.890 | 1.975 | -0.743 | 0.802 | 1.975 | 0.177 | 0.967 | 1.975 |
| Hypertensive nephropathy | 3.984 | 0.222 | 1.636 | -0.097 | 0.971 | 1.636 | 1.128 | 0.663 | 1.636 | 0.908 | 0.724 | 1.636 | 6.803 | 0.012 | 1.636 | -1.108 | 0.697 | 1.636 | 1.523 | 0.712 | 1.636 |
| Other causes | 2.804 | 0.411 | 1.548 | 0.020 | 0.994 | 1.548 | 0.827 | 0.760 | 1.548 | -0.056 | 0.983 | 1.548 | 3.827 | 0.173 | 1.548 | -2.295 | 0.441 | 1.548 | -2.646 | 0.539 | 1.548 |
| Chronic glomerulonephritis | 0 |  |  | 0 |  |  | 0 |  |  | 0 |  |  | 0 |  |  | 0 |  |  | 0 |  |  |
| **Comorbidities** |  | | |  | | |  | | |  | | |  | | |  | | |  | | |
| Hyperlipidemia | -11.990 | 0.002 | 1.519 | -4.871 | 0.117 | 1.519 | -0.849 | 0.777 | 1.519 | -2.024 | 0.497 | 1.519 | -2.250 | 0.469 | 1.519 | -3.699 | 0.263 | 1.519 | 0.039 | 0.993 | 1.519 |
| Ischemic heart disease | 3.494 | 0.311 | 1.362 | 2.280 | 0.420 | 1.362 | 4.722 | 0.085 | 1.362 | 5.033 | 0.065 | 1.362 | 1.526 | 0.590 | 1.362 | 4.454 | 0.140 | 1.362 | 4.832 | 0.268 | 1.362 |
| Diabetes mellitus | -4.586 | 0.158 | 1.389 | 2.135 | 0.423 | 1.389 | -0.822 | 0.749 | 1.389 | -0.952 | 0.710 | 1.389 | -2.716 | 0.309 | 1.389 | 5.078 | 0.074 | 1.389 | -0.590 | 0.886 | 1.389 |
| Other comorbidities | -1.726 | 0.706 | 1.254 | 7.933 | 0.035 | 1.254 | 4.468 | 0.220 | 1.254 | 4.872 | 0.178 | 1.254 | 4.832 | 0.201 | 1.254 | 3.320 | 0.407 | 1.254 | -3.968 | 0.493 | 1.254 |
| Hypertension | 0 |  |  | 0 |  |  | 0 |  |  | 0 |  |  | 0 |  |  | 0 |  |  | 0 |  |  |
| **Presence of comorbidities** |  | | |  | | |  | | |  | | |  | | |  | | |  | | |
| No | -4.737 | 0.134 | 1.740 | -8.593 | <0.001 | 1.740 | -3.769 | 0.133 | 1.740 | -5.659 | 0.024 | 1.740 | 2.625 | 0.313 | 1.740 | -5.176 | 0.061 | 1.740 | -1.324 | 0.740 | 1.740 |
| Yes | 0 |  |  | 0 |  |  | 0 |  |  | 0 |  |  | 0 |  |  | 0 |  |  | 0 |  |  |
| **Complications** |  | | |  | | |  | | |  | | |  | | |  | | |  | | |
| CKD-MBD | 0.604 | 0.844 | 1.502 | 3.907 | 0.122 | 1.502 | 1.896 | 0.437 | 1.502 | 1.747 | 0.471 | 1.502 | 1.495 | 0.555 | 1.502 | 3.102 | 0.249 | 1.502 | 6.287 | 0.106 | 1.502 |
| Renal hypertension | -1.069 | 0.689 | 1.492 | 1.836 | 0.402 | 1.492 | 0.670 | 0.752 | 1.492 | 4.658 | 0.028 | 1.492 | 2.897 | 0.188 | 1.492 | 1.803 | 0.440 | 1.492 | 0.824 | 0.807 | 1.492 |
| Malnutrition | -0.763 | 0.825 | 1.123 | -0.700 | 0.805 | 1.123 | 2.794 | 0.308 | 1.123 | 2.661 | 0.329 | 1.123 | 2.525 | 0.375 | 1.123 | -0.679 | 0.822 | 1.123 | 1.602 | 0.714 | 1.123 |
| Intradialytic hypotension | -4.362 | 0.261 | 1.411 | 0.411 | 0.897 | 1.411 | -2.056 | 0.503 | 1.411 | -0.322 | 0.916 | 1.411 | 2.620 | 0.411 | 1.411 | 2.690 | 0.427 | 1.411 | -0.156 | 0.975 | 1.411 |
| Hyperuricemia | 6.226 | 0.121 | 1.482 | 2.762 | 0.401 | 1.482 | 1.472 | 0.643 | 1.482 | 4.540 | 0.151 | 1.482 | 2.688 | 0.415 | 1.482 | -2.794 | 0.425 | 1.482 | 6.877 | 0.175 | 1.482 |
| Other complications | 8.028 | 0.169 | 1.244 | 3.028 | 0.527 | 1.244 | -5.997 | 0.195 | 1.244 | 10.523 | 0.023 | 1.244 | 6.101 | 0.204 | 1.244 | 2.712 | 0.595 | 1.244 | 5.571 | 0.450 | 1.244 |
| Renal anemia | 0 |  |  | 0 |  |  | 0 |  |  | 0 |  |  | 0 |  |  | 0 |  |  | 0 |  |  |
| **Polypharmacy status** |  | | |  | | |  | | |  | | |  | | |  | | |  | | |
| Polypharmacy | 2.403 | 0.434 | 2.003 | -1.220 | 0.628 | 2.003 | 1.070 | 0.660 | 2.003 | -0.923 | 0.703 | 2.003 | 2.352 | 0.352 | 2.003 | -1.177 | 0.660 | 2.003 | -4.535 | 0.242 | 2.003 |
| Excessive polypharmacy | 3.085 | 0.431 | 2.537 | 0.655 | 0.838 | 2.537 | 5.144 | 0.098 | 2.537 | -0.818 | 0.791 | 2.537 | -3.499 | 0.277 | 2.537 | -1.497 | 0.661 | 2.537 | -5.982 | 0.227 | 2.537 |
| Non-polypharmacy | 0 |  |  | 0 |  |  | 0 |  |  | 0 |  |  | 0 |  |  | 0 |  |  | 0 |  |  |
| R^2^ | 0.378 | | | 0.297 | | | 0.469 | | | 0.429 | | | 0.342 | | | 0.256 | | | 0.229 | | |
| Durbin–Watson | 2.152 | | | 2.019 | | | 2.164 | | | 2.103 | | | 1.816 | | | 2.122 | | | 1.843 | | |

VIF—Variance Inflation Factor
